# Interactions of Prototype Foamy Virus Capsids with Host Cell Polo-Like Kinases Are Important for Efficient Viral DNA Integration

**DOI:** 10.1371/journal.ppat.1005860

**Published:** 2016-08-31

**Authors:** Irena Zurnic, Sylvia Hütter, Ute Rzeha, Nicole Stanke, Juliane Reh, Erik Müllers, Martin V. Hamann, Tobias Kern, Gesche K. Gerresheim, Fabian Lindel, Erik Serrao, Paul Lesbats, Alan N. Engelman, Peter Cherepanov, Dirk Lindemann

**Affiliations:** 1 Institute of Virology, Medical Faculty "Carl Gustav Carus", Technische Universität Dresden, Dresden, Germany; 2 CRTD/DFG-Center for Regenerative Therapies Dresden, Technische Universität Dresden, Dresden, Germany; 3 Department of Cancer Immunology and Virology, Dana-Farber Cancer Institute, Boston, United States of America; 4 Clare Hall Laboratories, The Francis Crick Institute, South Mimms, United Kingdom; Fred Hutchinson Cancer Research Center, UNITED STATES

## Abstract

Unlike for other retroviruses, only a few host cell factors that aid the replication of foamy viruses (FVs) via interaction with viral structural components are known. Using a yeast-two-hybrid (Y2H) screen with prototype FV (PFV) Gag protein as bait we identified human polo-like kinase 2 (hPLK2), a member of cell cycle regulatory kinases, as a new interactor of PFV capsids. Further Y2H studies confirmed interaction of PFV Gag with several PLKs of both human and rat origin. A consensus Ser-Thr/Ser-Pro (S-T/S-P) motif in Gag, which is conserved among primate FVs and phosphorylated in PFV virions, was essential for recognition by PLKs. In the case of rat PLK2, functional kinase and polo-box domains were required for interaction with PFV Gag. Fluorescently-tagged PFV Gag, through its chromatin tethering function, selectively relocalized ectopically expressed eGFP-tagged PLK proteins to mitotic chromosomes in a Gag STP motif-dependent manner, confirming a specific and dominant nature of the Gag-PLK interaction in mammalian cells. The functional relevance of the Gag-PLK interaction was examined in the context of replication-competent FVs and single-round PFV vectors. Although STP motif mutated viruses displayed wild type (wt) particle release, RNA packaging and intra-particle reverse transcription, their replication capacity was decreased 3-fold in single-cycle infections, and up to 20-fold in spreading infections over an extended time period. Strikingly similar defects were observed when cells infected with single-round wt Gag PFV vectors were treated with a pan PLK inhibitor. Analysis of entry kinetics of the mutant viruses indicated a post-fusion defect resulting in delayed and reduced integration, which was accompanied with an enhanced preference to integrate into heterochromatin. We conclude that interaction between PFV Gag and cellular PLK proteins is important for early replication steps of PFV within host cells.

## Author Summary

Viruses are masters at exploiting host cell machineries for their replication. For human immunodeficiency virus type 1 (HIV-1), the best-studied representative of the *Orthoretrovirinae* subfamily from the genus lentiviruses, numerous important virus-host interactions have been described. In contrast, only a few cellular proteins are known to influence the replication of foamy viruses (FVs, also known as spumaviruses), an intriguing type of complex retrovirus of the *Spumaretrovirinae* subfamily that combines features of both retroviruses and hepadnaviruses in its replication strategy. Given the increasing interest in FVs as gene transfer tools and their unique status within the retrovirus family, this discrepancy urged the identification of novel host cell interaction partners of FV structural components. This study focused on prototype FV (PFV), the best-characterized member of FVs, and its capsid protein, Gag, as the central player of viral replication. Members of the mitosis-regulatory, polo-like kinase (PLK) family were identified as novel Gag binding partners. The Gag interaction with PLK1 (and possibly also PLK2) facilitated efficient PFV genome integration into host chromatin, ensuring successful replication and viral spread in infected target cell cultures. Collectively, our results elucidate the first link between cell cycle regulatory networks and the mitosis-dependent PFV integration process.

## Introduction

As obligatory intracellular parasites, retroviruses depend on host cell machineries for successful replication. Over the years, many cellular proteins counteracting or aiding retroviral replication have been identified [reviewed in 1, 2]. Expectedly, a large number of these factors are capsid protein interaction partners [reviewed in 3, 4, 5]. Since retroviral Gag proteins orchestrate numerous intracellular trafficking steps and viral budding, they naturally recruit numerous cellular factors and machineries to accomplish their functions.

Similar to its orthoretroviral relatives, prototype foamy virus (PFV), the best-studied member of the genus spumavirus in the *Spumaretrovirinae* subfamily, relies on Gag interactions with host cell proteins for successful replication [reviewed in 6]. However, unlike for orthoretroviruses such as human immunodeficiency virus type 1 (HIV-1), few PFV Gag interaction partners have been identified to date. This is surprising, given the peculiarities of the PFV replication cycle and their significance to understanding retroviral evolution and vector development [reviewed in 7, 8].

To our knowledge, only six host factors that interact either directly or indirectly with PFV Gag have been identified, but given the multitude of roles PFV Gag accomplishes for viral replication in cells, this list is clearly far from complete. The tumor susceptibility gene 101 (Tsg101) component of the ESCRT-I complex directly interacts with primate FV Gag proteins to promote efficient virus budding [9, 10]. Furthermore, direct or indirect interactions with actin and core histones (H2A and H2B) were identified upon co-precipitation of these cellular proteins with ectopically expressed PFV Gag [11, 12]. These interactions are thought to be involved in PFV Gag precursor protein processing regulation and facilitate docking of the PFV pre-integration complex (PIC) to host cell chromosomes, respectively. FVs are also known to be restricted in a species-specific manner by cellular Trim5α proteins [13–15]. The viral determinants essential for restriction were mapped to the N-terminus of FV Gag, which probably engages Trim5α in the context of the viral capsid. In addition, a member of the DEAD-box helicase family, DDX6, was found to co-localize with PFV Gag at assembly sites and aid in viral RNA encapsidation into newly assembling FV particles [16]. Finally, a potentially direct interaction of dynein light chain 8 with a coiled-coil domain within the N-terminus PFV Gag was reported, which appears to facilitate transport of incoming capsids to the centrosome of host cells [17]. At the centrosome of non-dividing cells PFV capsids can remain intact and biologically active for several weeks [18]. PFV capsids seem to proceed only to further disassembly steps upon entry of the cell into mitosis, which is required for PFV genome integration into cellular chromatin. The cues and machineries coordinating viral uptake and intracellular trafficking are incompletely characterized. Until now, it has only been speculated that members of cell cycle regulatory pathways may aid these processes.

**Fig 1:**
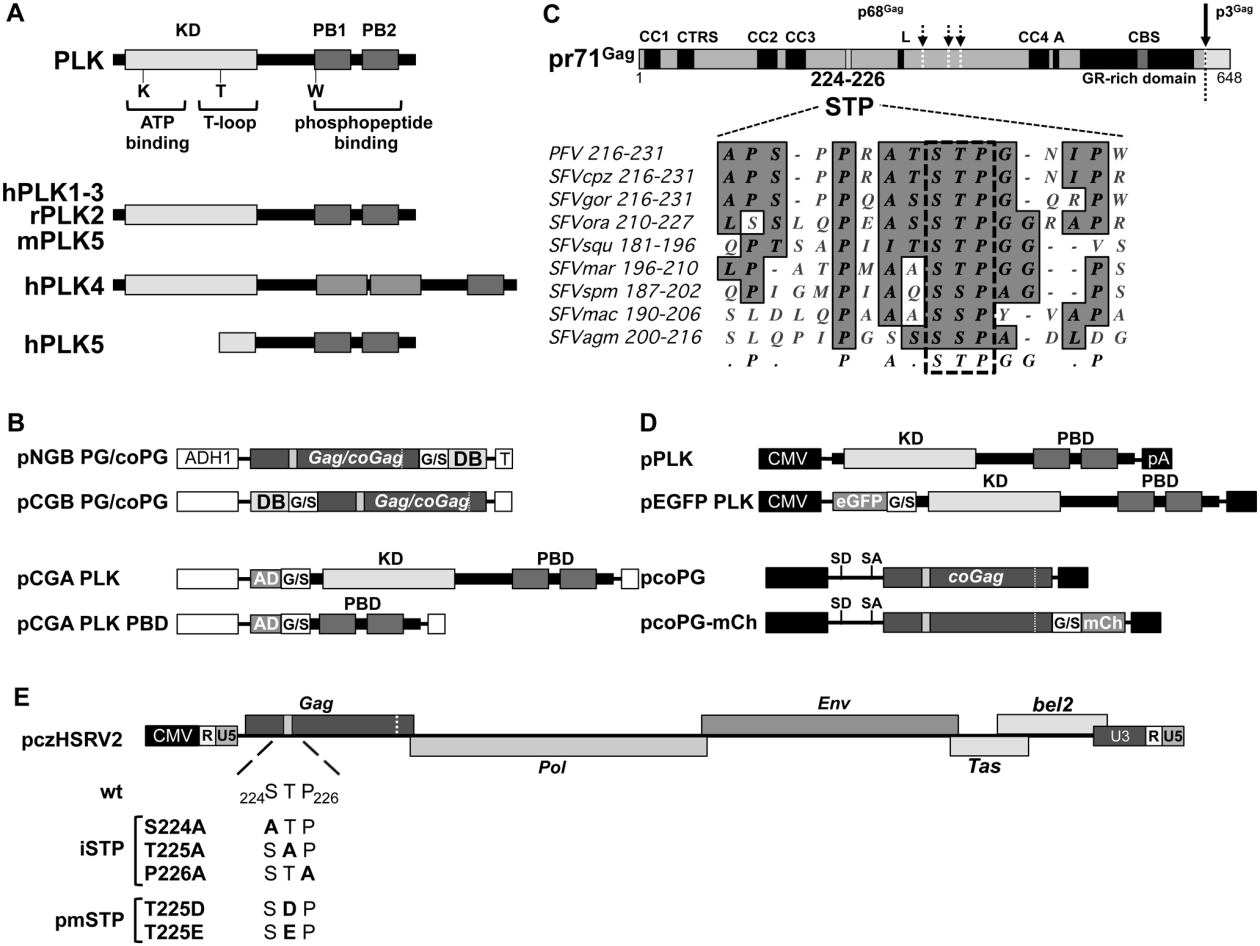
PFV Gag and PLK constructs utilized in this study. (A) The structures of human, rat and mouse PLK proteins addressed are schematically illustrated, highlighting the amino acids required for different functions (ATP-binding and hydrolysis; T-loop for kinase autoactivation; substrate S-pS/pT-P motif recognition and binding). KD: kinase domain; PB1-2: polo boxes 1 and 2 (comprising the PB domain (PBD)). (B) Schematic outline of the yeast PFV Gag- and PLK-encoding expression constructs. (C) Schematic representation of full-length PFV Gag, with functional domains and the central S_224_-T_225_-P_226_ motif highlighted. The alignment with the central STP or SSP motives of other primate foamy virus (simian FV; SFV) Gag proteins is shown below. The origin of SFV isolates is noted as follows, cpz: chimpanzee; gor: gorilla; ora: orangutan; squ: squirrel monkey; mar: marmoset; spm: spider monkey; mac: macaque; agm: African green monkey. Primary and secondary proteolytic cleavage sites in the PFV Gag protein are highlighted by full- and dashed arrows, respectively. (D) Schematic outline of the mammalian PFV Gag- and PLK-encoding expression constructs. (E) Schematic outline of proviral PFV expression constructs, highlighting the introduced STP motif amino acid exchanges. CC1-CC4: coiled-coil domains 1–4; CTRS: cytoplasmic targeting and retention signal; L: late domain; A: assembly domain; GR-rich domain: glycine-arginine-rich domain; solid vertical arrow: primary Gag processing site; dashed vertical arrows: secondary Gag processing sites; wt; wild type; Gag: authentic *gag* ORF; coGag; expression-optimized *gag* ORF; ADH1: yeast alcohol dehydrogenase 1 promoter; AD: GAL4 activation domain; DB: GAL4 DNA-binding domain; T: yeast terminator sequence; G/S: glycine serine linker; CMV: cytomegalovirus promoter; R: PFV long terminal repeat (LTR) repeat region; U5: PFV LTR unique 5’ region; U3: PFV LTR unique 3’ region; eGFP: enhanced green fluorescent protein; mCh: mCherry; pA: polyadenylation sequence; hPLK1-5: human PLKs1-5; rPLK2: rat PLK2; mPLK5: mouse PLK5.

One family of mitosis promoting proteins is the polo-like kinases (PLKs), which are direct downstream effectors of cyclin/cyclin-dependent kinase complex (Cyclin/CDK) cascades. PLK proteins are cellular Ser/Thr protein kinases that in vertebrates comprise five paralogues, PLK1-5 [reviewed in 19, 20, 21]. They are crucial regulators of cell cycle progression, centriole duplication, mitosis, cytokinesis and DNA damage response. However, other functions unrelated to cell cycle control such as protein aggregation and cellular stress response have also been attributed to various PLKs [22, 23]. Mammalian PLK proteins are characterized by a modular structure (Fig 1A), comprising an N-terminal (Ser/Thr) catalytic kinase domain (KD) and a C-terminal polo-box domain (PBD). The PBD, which harbors one or more polo-boxes (PBs) that coordinate substrate binding, regulates catalytic activities and subcellular localization of PLKs [24–26]. With the exception of PLK4, which contains a unique PBD organization [27], the PLK PBDs facilitate binding to phosphopeptides with an optimal Ser-(pThr/pSer)-Pro (S-(pT/pS)-P) substrate binding motif [28, 29]. The best-studied member of the PLK family is PLK1, which typically utilizes its PBD to target proteins that have been primed through phosphorylation at their PLK binding site by other cellular kinases, such as CDKs or MAPKs [29, 30]. However, there are a few cases known where the PLK1 PBD binds non-phosphorylated peptides or phosphopeptides that were previously phosphorylated by PLK1 itself [30, 31]. Thus, multiple mechanisms exist to modulate the function and interaction of PLK1 and other PLKs and their targets in space and time.

Although replication of various viruses is influenced by cellular PLKs and viruses have been reported to modify the activity of PLKs for their replication, only a few direct interactions of viral proteins with PLKs have been reported. For example the P protein component of the viral RNA dependent RNA polymerase (vRdRp) of paramyxoviruses, such as parainfluenza virus 5 (PIV5) or mumps virus (MuV), harbors a PLK1 STP binding site and is phosphorylated at another site by PLK1 [32, 33]. PLK1-mediated P protein phosphorylation downregulates viral gene expression and thereby avoids efficient induction of the host innate immune response [34]. Furthermore, the interaction of the E2 protein of human papillomavirus type 5 with PLK1 was reported to inhibit Brd4 phosphorylation by PLK1, thereby interfering with cellular functions of Brd4 in promoting cell cycle progression from the G1 to the S phases [35]. Finally, binding of PLK1 to the NS5A protein of hepatitis C virus and its hyperphosphorylation by PLK1 is important for efficient virus replication [36].

To gain further insight into the cellular biology of PFV replication and to identify new potential novel Gag interactors, we conducted a yeast two-hybrid (Y2H) screen using Gag protein as bait against different human cDNA expression libraries. In addition to the known PFV Gag interactor Tsg101, a member of the PLK family was identified as a new hit. A detailed characterization and analysis revealed that PFV Gag specifically interacts with cellular PLK1 and PLK2 and alters their intracellular distribution. PFV particles, unable to interact with cellular PLKs upon virus entry, display an infectivity defect manifested by a delayed and reduced capacity to integrate viral genomic DNA (vgDNA) into host cell chromatin.

## Results

### Mammalian polo-like kinases are novel PFV Gag interactants

To elucidate intracellular steps of PFV replication in more detail, we performed a Y2H screen, aiming to identify novel Gag protein interaction partners. Full-length (FL) PFV Gag (PG) was used as bait, fused either to the N- or C-terminus of the GAL4 DNA binding domain (DB, here referred to as pNGB PG/coPG and pCGB PG/coPG, respectively) (Fig 1B). These bait constructs were used and tested for interaction with a normalized HeLa cDNA expression library and two different full-length human open reading frame (ORF) expression libraries, where prey proteins were fused to the C-terminus of the GAL4 activation domain (AD, pGADT7, here referred to as pCGA). Several human proteins were identified as likely PFV Gag interactors. Among them was the known PFV Gag interactor, Tsg101 (7 different fragments, identified a total of 51 times), previously shown to interact with PFV Gag in Y2H experiments [10]. A potential new PFV Gag interactor identified multiple times in the screen was human PLK2 (hPLK2; 7 different fragments spanning the C-terminal PBD, identified a total of 20 times). We note that the hPLK2 PBD scored in the assay only in combination with the C-terminally tagged FL PFV Gag protein bait (pNGB PG/coPG), whereas Tsg101 was identified with both PFV Gag baits.

Additional Y2H experiments were performed to validate the results, investigate whether PFV can interact with other members of the PLK family, and characterize essential interaction domains/motifs within both proteins. The analyses included various bait constructs encoding FL PFV Gag as well as point mutants or N- and C-terminal truncations, fused to the N- or C-terminus of the GAL4 DB (Fig 1B). Prey constructs encoded FL ORFs of hPLK1-3, 5, mouse PLK5 (mPLK5), rat PLK2 (rPLK2) and point mutants thereof, as well as the PBDs of hPLK1-4, fused to the C-terminus of the GAL4 AD (pCGA-PLKs) (Fig 1A and 1B). A prey encoding the FL Tsg101 ORF was utilized as positive control, whereas negative controls included the empty bait (pNGB, pCGB) and prey (pCGA, pNGA) vectors.

**Fig 2:**
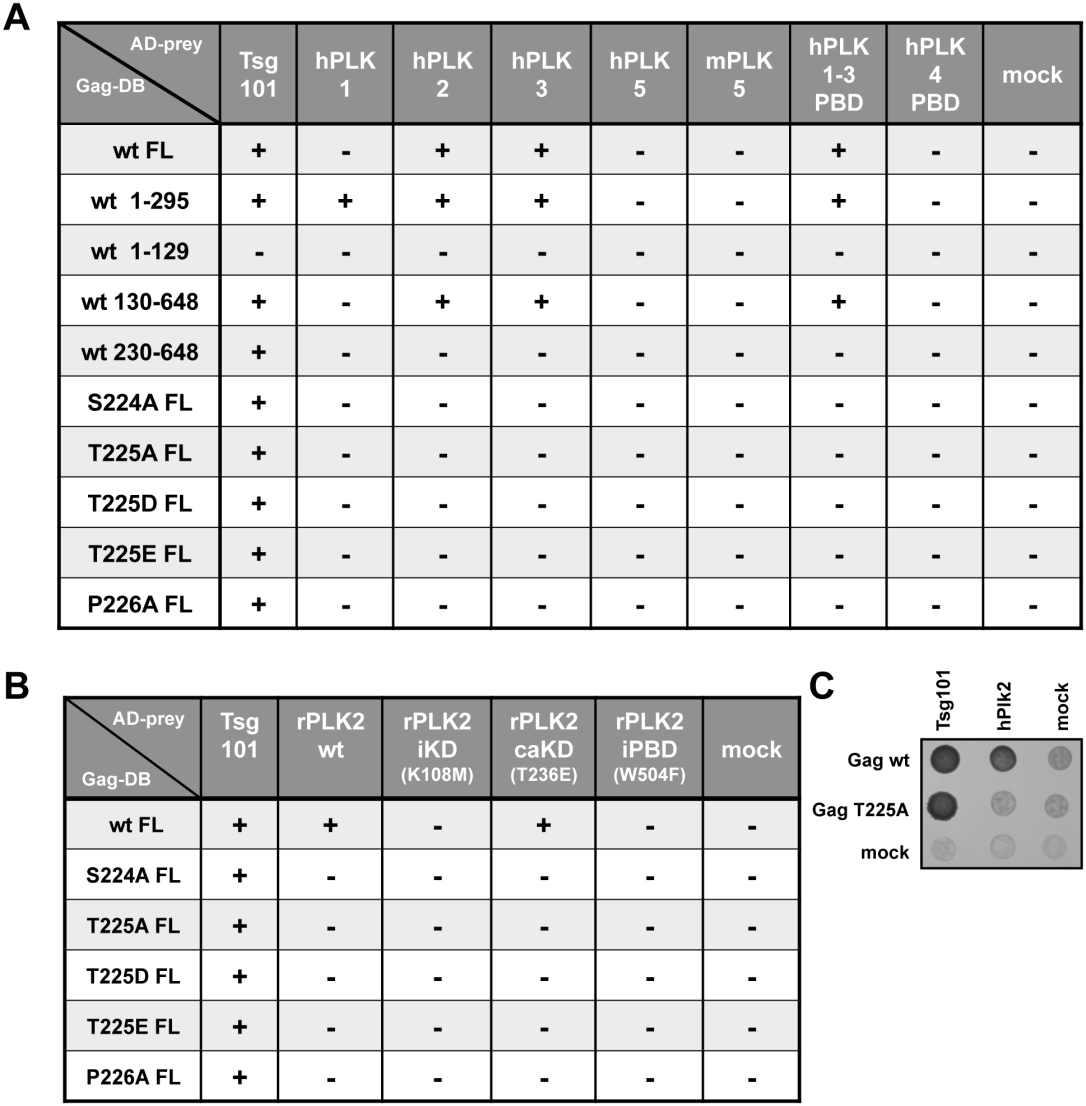
Y2H analysis of PFV Gag-PLK interactions. Different variants of the PFV Gag protein (full length (FL), indicated truncations and inactivating (iSTP) or phosphomimetic (pmSTP) point mutants) were tested for interaction with human (hPLK), mouse (mPLK) and rat PLK proteins (rPLK) or, where indicated, respective PBDs. PFV Gag was provided fused to the GAL4 DB (Gag-DB) in combination with Tsg101- or PLK proteins fused to GAL4 AD (AD-Prey). Presence and absence of interaction between each partner is marked by either “+” or “-“, respectively. Data of n = 4 independent experiments are summarized. (A) Results of PFV Gag interaction with human and mouse PLK proteins. (B) Results of PFV Gag interaction with rPLK2 variants. (C) Readout system of experimental results, assessing transformed yeast growth on selective media, exemplified by DB-Gag wt, DB-Gag T225A or empty bait in combination with AD-Tsg101, AD-hPLK2 or empty prey. iKD: inactive kinase domain; caKD: constitutively active kinase domain; iPBD: inactive polo-box domain.

The results of the Y2H analysis are summarized in Fig 2 and S1 Fig FL PFV Gag interacted with FL hPLK2 and 3, rPLK2 as well as PBDs of hPLK1-3 (Fig 2A and 2B). FL PFV Gag did not interact with FL hPLK1, though PFV Gag baits with C-terminal deletions of 104 or more residues did interact with hPLK1 (Fig 2A; S1A and S1B Fig). Interactions with other tested PLK proteins were not detected (Fig 2; S1 Fig). These results indicate that FL PFV Gag can interact with various hPLKs and that these interactions are mediated via the kinase PBDs. The use of N- and C-terminal Gag bait truncations allowed characterization of a minimal essential interaction domain spanning amino acids 130–295 (Fig 2A; S1 Fig). A closer inspection of this region revealed the presence of a Ser-Thr-Pro (S-T-P) motif at position 224–226, which was unique within the FL PFV Gag protein (Fig 1C). Interestingly, this motif, which matches the minimal consensus PLK recognition and binding site (S-T/S-P) [29], is present in all primate FV Gag proteins (Fig 1C). Exchange of any of the residues of the PFV Gag STP motif in context of the FL protein, including inactivating (iSTP) and phosphomimetic (pmSTP) mutations at T225, abolished interactions with all PLK preys that interacted with the wild type (wt) Gag protein, without affecting the interaction with Tsg101 (Fig 2A and 2B). Hence, a preserved PFV Gag STP motif seems to be essential for interaction with cellular PLK proteins.

In order to determine which domains and enzymatic properties of the PLK proteins are important for the interactions with PFV Gag, we focused on FL rPLK2 ORFs with point mutations in critical residues as preys (Fig 2B; S1C Fig). As expected, none of the iSTP or pmSTP Gag constructs interacted with any of these rPLK2 variants, confirming interaction dependency on the PFV Gag STP motif. FL wt Gag protein interacted with wt and constitutively active T236E (caKD) rPLK2, whereas no interaction was detected with kinase inactive K108M (iKD) rPLK2 or its PBD mutant W504F (iPBD) (Fig 2B). These results suggest that the kinase activity of rPLK2 is required for stable interaction with FL PFV Gag, which is mediated by the rPLK2 PBD.

Examination of the amino acid sequence of the other PFV structural proteins revealed the presence of two additional consensus STP peptide motifs in Pol but none in the cytoplasmic domains of Env. Both putative Pol PLK binding sites are located in the integrase domain of Pol surrounding T961 and T1058 (S2A Fig). However, an extensive Y2H interaction analysis using bait constructs expressing either a PFV Pol precursor protein with enzymatically inactive PR or the mature integrase subunit failed to demonstrate signs of interaction with any of the PLK prey constructs that showed interactions with PFV Gag (S2B Fig). Principal protein interaction functionality of the employed Pol and integrase bait constructs was demonstrated through their positive interaction, probably as a consequence of Pol/integrase oligomerization when using respective bait and prey construct combinations (S2B Fig).

### PFV Gag co-localizes with mammalian PLK1 and PLK2

**Fig 3:**
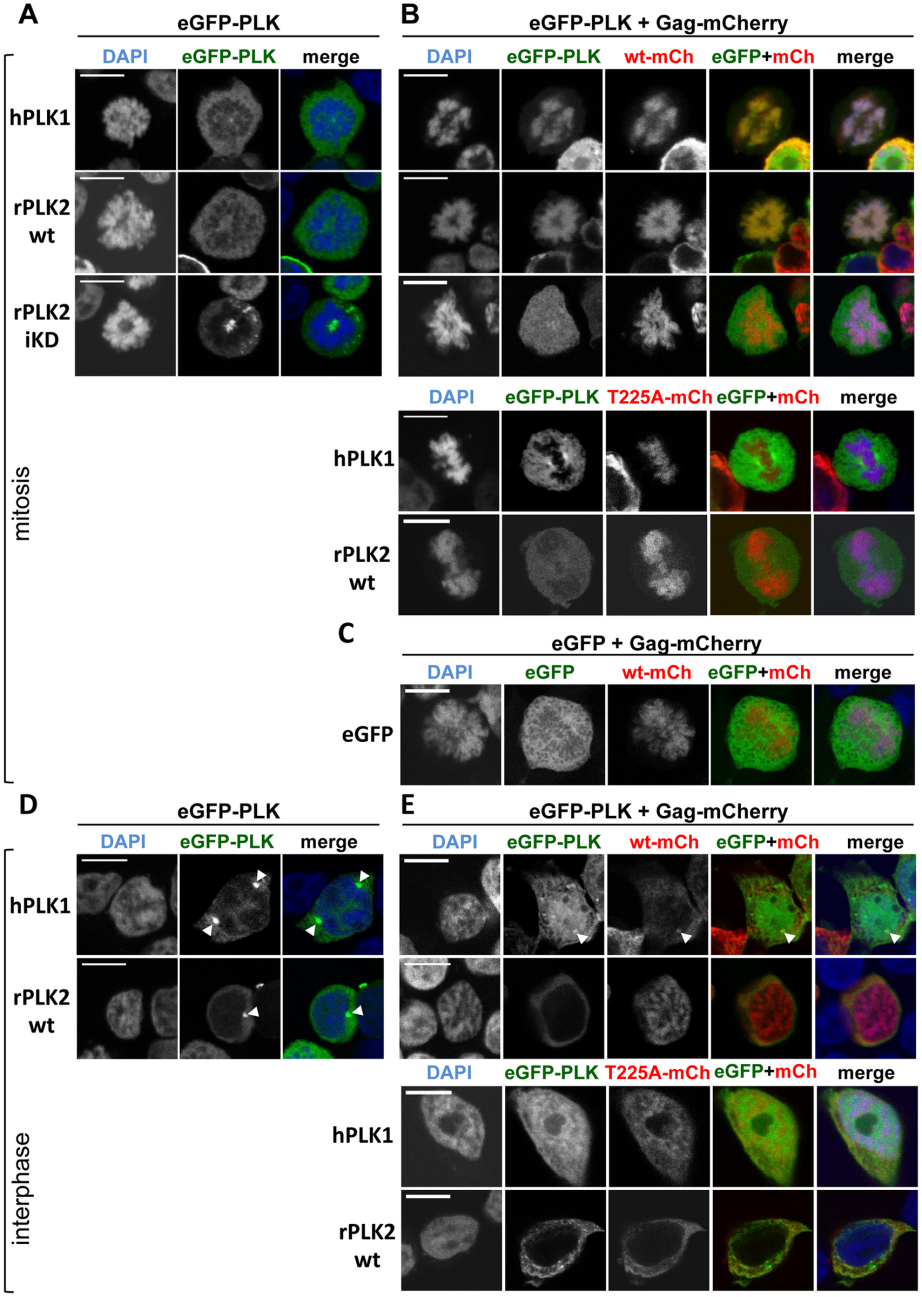
Localization of ectopically-expressed, fluorescently-tagged PFV Gag and PLK proteins in mammalian cells. eGFP-PLK-expressing constructs alone (left panels) or a combination of eGFP or eGFP-PLK and Gag-mCherry encoding expression constructs (right panels) were transfected into 293T cells, as indicated above each panel of images. Forty-eight hours post-transfection, protein localization patterns were examined in fixed cells by confocal laser scanning microscopy (CLSM). Channels of the individual fluorescence micrographs are indicated on top, and the PLK variant used is indicated on the left. White arrowheads indicate fluorescent PLK foci presumed to be centrosomes. Data are representative of n = 5 independent experiments. (A) Localization patterns of eGFP-tagged PLK proteins (detected in eGFP-PLK channel) in mitotic cells transfected with the corresponding constructs. (B) Localization patterns of eGFP-tagged PLK and mCherry-tagged Gag proteins detected in corresponding channels (Gag variant used labeled either as wt-mCh or T225A-mCh) in mitotic cells. (C) Localization of eGFP and wt Gag-mCherry in mitotic cells. (D) Localization patterns of eGFP-tagged PLK proteins (detected in eGFP-PLK channel) in interphase cells transfected with the corresponding constructs. (E) Localization patterns of eGFP-tagged PLK and mCherry-tagged Gag proteins detected in corresponding channels (Gag variant used labeled either as wt-mCh or T225A-mCh) in interphase cells. Scale bar: 10 μm.

To confirm the interaction between Gag and PLK1-3 in human cells, we investigated localization patterns of ectopically expressed, fluorescently labeled proteins. Constructs encoding C-terminally mCherry-tagged FL PFV Gag proteins (either wt or iSTP/pmSTP variants) and N-terminally eGFP-tagged variants of either FL hPLK1-3 or rPLK2 proteins were co-transfected into 293T cells. Representative results of confocal fluorescence microscopy analysis for selected Gag-PLK combinations are shown in Fig 3 and S3 Fig.

Introduction of single mutations into the PFV Gag STP motif had no effect on intracellular Gag localization. As described previously [37], all PFV Gag variants bound to chromatin during mitosis, completely overlapping the DAPI chromatin signal (Fig 3B and 3C). In line with previous observations, eGFP-tagged PLKs displayed speckled nuclear and diffuse cytoplasmic distributions when expressed on their own (Fig 3A and 3D; S3A Fig) [38]. Importantly, none of the PLKs showed the overall chromatin association that was observed for PFV Gag (Fig 3A and 3C; S3A Fig). Strikingly, the subcellular localization of hPLK1, 2 and rPLK2, and to less of an extent also hPLK3, was dramatically altered during mitosis in the presence of wt but not iSTP PFV Gag (Fig 3B; S3B and S3C Fig). Wt PFV Gag recruited PLK1 and PLK2 proteins to mitotic condensed chromatin resulting in an almost complete overlap of both signals, whereas for hPLK3 only a weak co-localization was observed. Distinct co-localization was not observed between any of the Gag variants with the examined eGFP-PLK fusions in interphase cells (Fig 3D and 3E; S3 Fig). In some cases, overlap of mCherry and eGFP signals was observed in a diffuse pattern throughout the cytoplasm of interphase cells co-transfected with wt Gag and hPLK1, hPLK2, or rPLK2 (Fig 3E; S3B Fig). Occasionally, co-localization between eGFP-hPLK1 and Gag-mCherry variants was noted at a perinuclear region, presumably the centrosome (Fig 3E, white arrow). However, this event could not be ascribed to a specific co-localization pattern, as it was independent of the functional Gag STP motif and both partners individually localized to the microtubule-organizing center (MTOC) (Fig 3D).

As these results established that an intact STP motif in PFV Gag was required for co-localization with hPLK1 and hPLK2 in human cells, we addressed next whether the kinase requirements would correspond to the Y2H data using rPLK2 as a representative candidate. Hence, the same set of rPLK2 variants involved in the Y2H interaction analysis was employed in combination with wt and T225A PFV Gag. Whereas wt Gag, but not its T225A variant, co-localized with wt (wt) and constitutively active rPLK2 (caKD), co-localization was not observed for any other rPLK2 protein variant including the kinase inactive (iKD) mutant (Fig 3B; S3D Fig).

Taken together, our results confirm that PLK1 and 2 and to a limited extent also PLK3 are cellular interaction partners of PFV Gag. The viral structural protein appears to recruit the PLKs to condensed, mitotic chromatin in an STP motif-dependent fashion, which requires functional PLK KD and PBDs.

### The putative PFV Gag PLK STP binding site is phosphorylated in released virions

**Fig 4:**
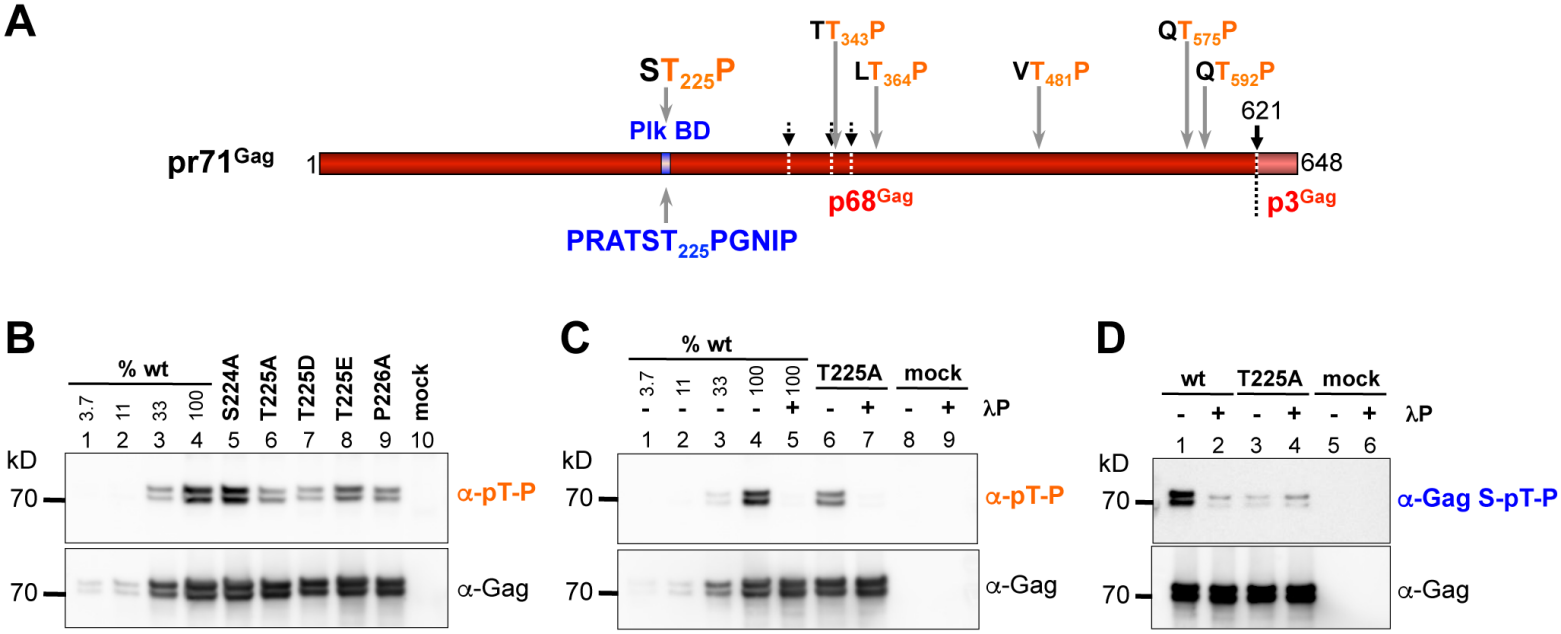
Analysis of PFV Gag phosphorylation status in purified virus particles. PFV virions were produced by transient transfection of the four-component PFV vector system, containing the pcoPG4 variants denoted above each of the blots, into 293T cells. Viral particles were pelleted from cell-free tissue culture supernatants by ultracentrifugation through 20% sucrose and equal amounts of particle lysates separated by SDS-PAGE were blotted to nitrocellulose membranes. The phosphorylation status of the particle-associated Gag variants was determined by the corresponding antibodies specific for different phosphorylated amino acid motives as indicated. Data are representative of n = 4 independent experiments. (A) Schematic illustration of PFV Gag protein organization with highlighted putative T-P motifs and surrounding amino acids recognized when phosphorylated at the threonine residue by either the α-pT-P (orange letters) or α-Gag S-pT-P (blue letters) phosphopeptide-specific antibody. Solid vertical arrow: primary Gag processing site; dashed vertical arrows: secondary Gag processing sites; (B) Detection of the phosphorylated Thr-Pro (pT-P) motives in PFV Gag wt, iSTP and pmSTP virus particles (α-pT-P antiserum) and comparison with the total Gag content in the particle lysates (α-Gag). (C) Comparison of the pT-P phosphorylation status in the wt and T225A PFV particles in the absence (-) or presence (+) of the Lambda Phosphatase (λP) pretreatment of viral proteins. (D) Comparison of the PFV Gag-associated S-T-P motif phosphorylation status (α-Gag S-pT-P; detected with the corresponding PFV Gag-specific antibody) in virus preparations containing either the wt or the T225A Gag variant in the absence (-) or presence (+) of the λP pretreatment.

The interaction of PLKs with substrates is reportedly strongly dependent on PLK binding site phosphorylation status [29]. Nonetheless, low affinity binding to non-phosphorylated binding sites, and a few binding sites phosphorylated by PLKs themselves, have been reported [reviewed in 39]. To determine the phosphorylation-status of the putative PFV Gag PLK STP binding site, we examined particle-associated Gag or protein immunoprecipitated from cell lysates with various phosphopeptide-specific antibodies, including a custom-made antiserum specific for the PFV Gag STP motif (Fig 4). Detectable amounts of phosphorylated Gag protein were absent in immunoprecipitates of lysates of 293T cells transiently transfected with expression constructs of the four-component PFV vector system. This suggests that the majority of cellular PFV Gag is not phosphorylated at threonine residues in the context of T-P motifs, including the PLK binding motif. In contrast, phosphorylated Gag was readily detected in lysates of released wt PFV virions pelleted by ultracentrifugation, by either a commercial pThr-Pro (pT-P)–specific monoclonal or the custom-made PFV Gag PLK binding site phospho-specific (Gag S-pT-P) polyclonal antibody (Fig 4B–4D). pT-P-specific signal intensity was reduced in particle lysate samples of all iSTP/pmSTP mutants, except S224A, which theoretically can still be phosphorylated at T225 by a non-PLK cellular priming kinase (Fig 4B). Furthermore, pT-P-specific signals were almost undetectable in both wt and T225A mutant particle lysates pretreated with phosphatase (Fig 4C). Hence, T225 phosphorylation appears to contribute to a large extent to the PFV Gag pT-P-specific signal detected in wt PFV particle samples. This was further substantiated by results using the custom-made PFV Gag S-pT-P-specific antiserum. A strong signal was detected in wt PFV particle samples, which was largely reduced by phosphatase pretreatment down to the background levels that were observed in PFV Gag T225A particle samples regardless of phosphatase treatment (Fig 4D).

In concordance with the results of Western blotting, mass spectrometric analysis revealed that a fraction of the PFV Gag protein recovered from SDS-PAGE-separated wt PFV particle samples is phosphorylated at the 223-TST-225 cluster (S4A and S4B Fig). Obtained high-resolution fragmentation spectra do not exclude that within the 223–225 cluster the phosphogroup is localized at the position 225 (S4C Fig).

Taken together, these results indicate the presence of significant amounts of PFV Gag protein with phosphorylated PLK binding site motif in released wt PFV virions, which can potentially serve as a high affinity interaction partner for cellular PLK proteins upon PFV entry into newly infected host cells.

### PFV Gag iSTP/pmSTP mutant particles have a replication defect

The interaction between PFV Gag and human PLK proteins has not been implicated in previous studies, but may be of importance for the cell cycle-dependent replication characteristics of PFV [40–42]. Hence, we investigated whether introduction of Gag iSTP/pmSTP variants into PFV particles may exert an effect on virus replication. The infectivity of the PFV Gag mutants was assessed in the context of both replication-competent and single-round PFV expression systems, with similar results obtained between systems.

**Fig 5:**
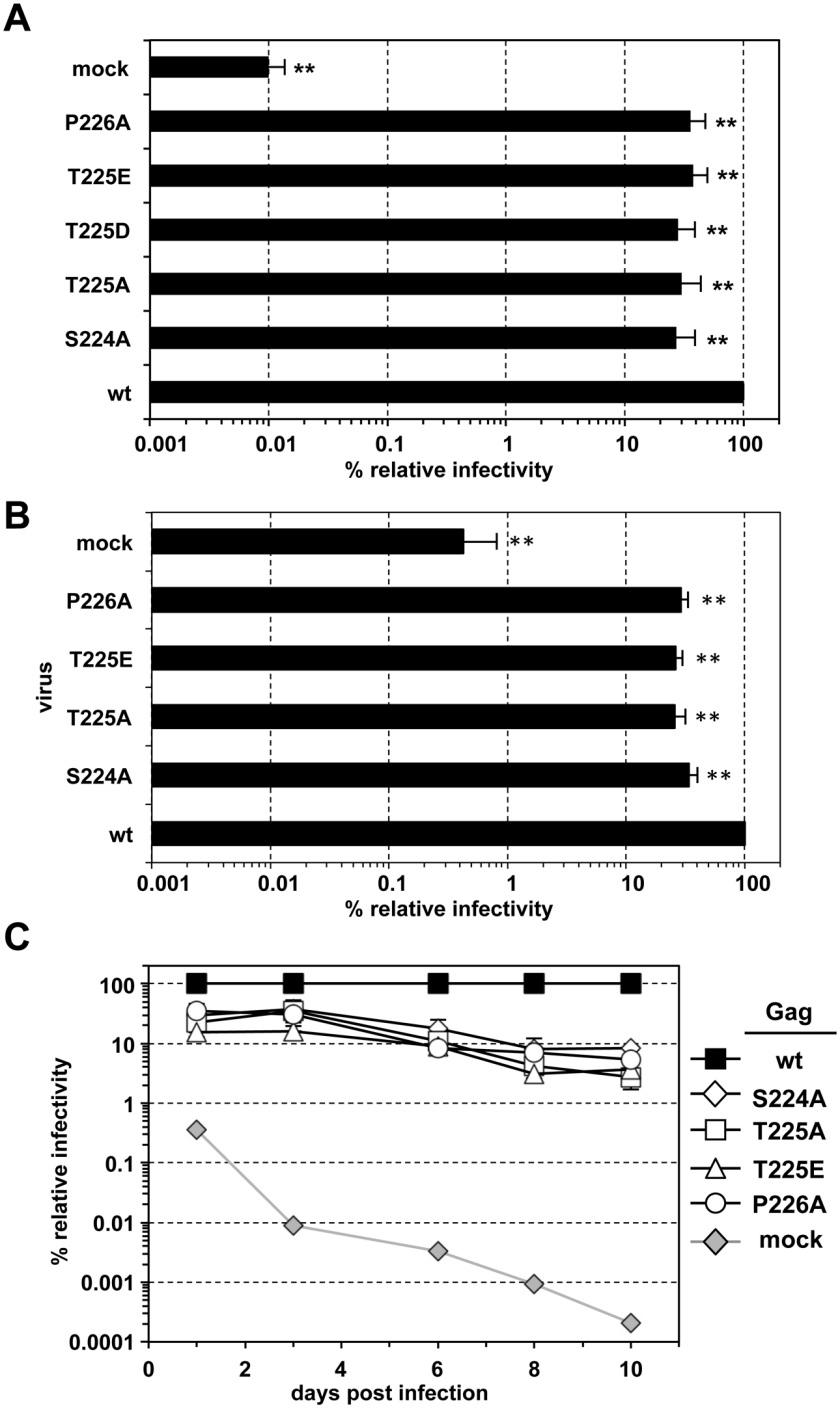
Analysis of PFV wt, iSTP- and pmSTP virions in single-round- and multiple-round infection experiments. (A) PFV virions were produced by transient transfection of 293T cells with the four-component PFV vector system, containing either the wt Gag or one of the denoted iSTP- and pmSTP Gag variants. Titers of harvested viruses were determined by flow cytometry analysis of infected HT1080 target cells three days post-infection. The mean values and standard deviation for each supernatant were calculated from samples of cells infected with serial virus dilutions as described in Material and Methods. The values obtained using wt PFV Gag expression plasmids were arbitrarily set to 100%. Relative means and standard deviations normalized for Gag content (except mock) from independent experiments (n = 4–9) are shown. Differences between means of wt virus and the individual mutants were analyzed by Welch’s t test (**, p<0.01). Absolute titers of wt supernatants ranged between 1.2 x 10^6^ and 1.2 x 10^7^ eGFP ffu/ml. (B) Replication-competent PFV virions were produced by transient transfection of proviral expression vectors, containing either the wt Gag or one of the denoted iSTP- and pmSTP Gag variants into 293T cells. Viruses were harvested two days post-transfection and used to infect HT1080 PLNE target cells. Titers were determined by flow cytometry analysis one day post-infection. The values obtained using wt PFV Gag expression plasmids were arbitrarily set to 100%. Relative means and standard deviations normalized for Gag content (except mock) from independent experiments (n = 3–8) are shown. Differences between means of wt virus and the individual mutants were analyzed by Welch’s t test (**, p<0.01). Absolute titers of wt supernatants ranged between 1.7 x 10^4^ and 7 x 10^4^ eGFP ffu/ml. (C) Titers of iSTP- and pmSTP mutant PFV particles relative to wt over multiple rounds of target cell infection. Viruses were produced and harvested as described in panel B and Gag content normalized amounts of viral supernatants were used to infect HT1080 PLNE in serial dilutions. At different time points post-infection (as indicated on the x-axis) cells were harvested for flow cytometry analysis to determine viral titers. The values obtained using wt PFV supernatants at each time point were arbitrarily set to 100%. Relative means and standard deviations from two independent experiments are shown.

Inclusion of any of the tested STP Gag mutations into the packaging construct decreased infectivity of respective single-round PFV vector supernatants on HT1080, human primary fibroblast MRC-5 cells or murine embryonic fibroblasts by 50–70% (Fig 5A; S5A Fig). A similar infectivity defect was observed early after target cell infection for replication-competent mutant viral particle preparations generated from full-length proviral expression constructs (Fig 5B). Interestingly, the replication defect of the Gag STP motif mutant viruses became more prominent upon prolonged growth of the infected cell cultures (Fig 5C). The infectivity of iSTP/pmSTP Gag-containing particles progressively mitigated down to 2 to 8% relative to wt after 10 days of co-cultivation. A similar effect was also observed in human primary fibroblast MRC-5 cells (S5B Fig), indicating the phenotype is not cell-type specific. Thus, PFV particles harboring Gag unable to interact with cellular PLKs display a diminished infectivity in single-round infections and fail to efficiently spread in target cell cultures of different origin.

### PFV Pol iSTP mutant particles show wt replication characteristics

Even though we were unable to detect any signs of PFV Pol interaction with cellular PLKs in the Y2H analysis, we nevertheless examined the influence of various PFV Pol STP motif mutants on viral infectivity in combination with either wt or iSTP mutant PFV Gag (S2C Fig). Unlike Gag iSTP mutant PFV particles, the Pol iSTP mutant particles neither showed a significant difference in viral infectivity nor did they further promote the Gag iSTP infectivity defect (S2C Fig). Taken together with the Y2H interaction analysis, these results strongly suggest that PFV Pol STP motifs located in the integrase domain did not mediate interactions with cellular PLKs and are dispensable for PFV-mediated infection of target cells.

### Gag iSTP mutant particles have wt-like assembly and particle release characteristics

**Fig 6:**
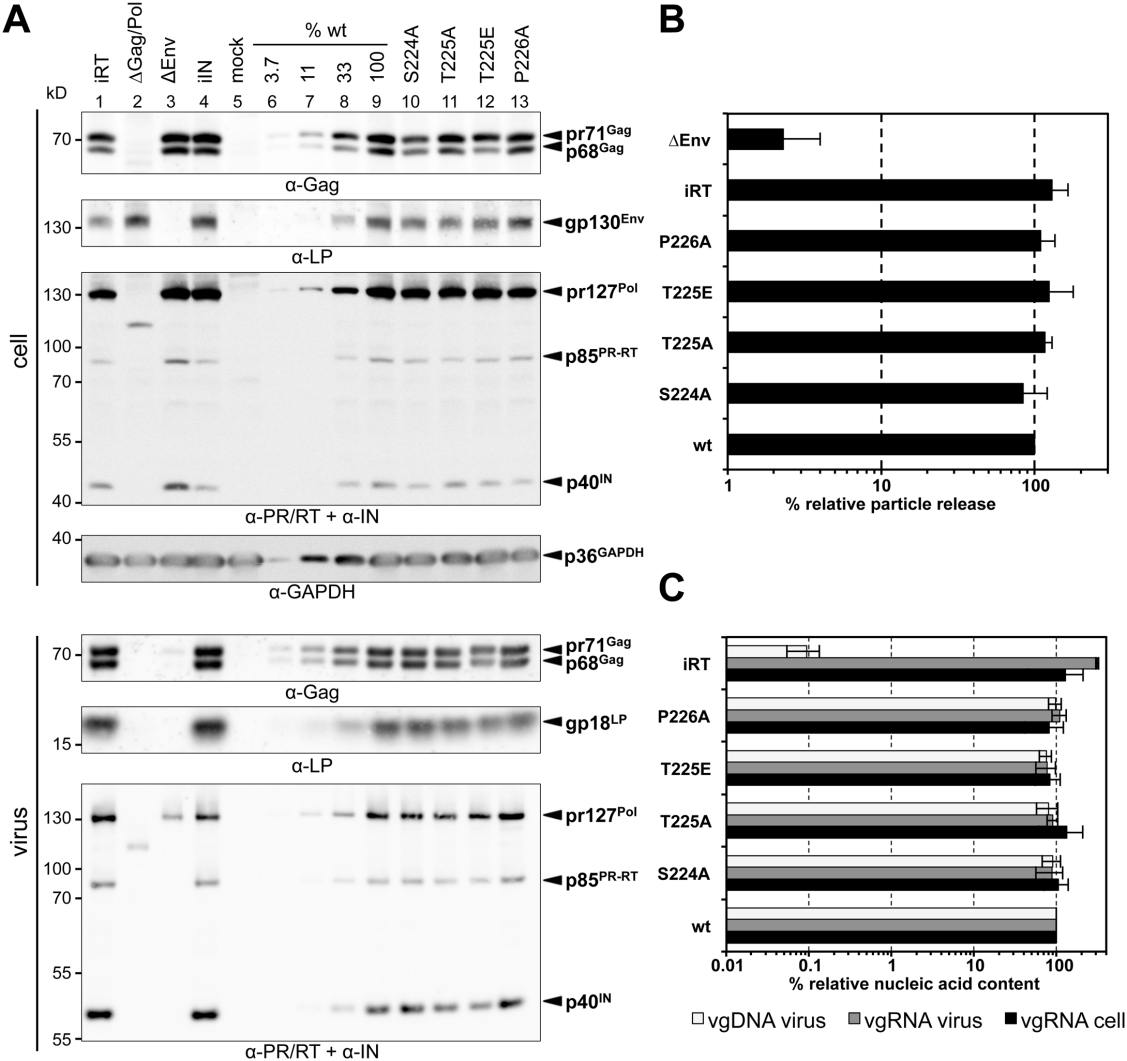
Biochemical characterization of the PFV production levels, particle release and nucleic acid contents in producer cells and released virions. Replication-competent PFV virions were produced by transient transfection of proviral expression constructs, containing either the wt Gag (wt) or one of the denoted iSTP- (S224A, T225A, P226A) or pmSTP (T225E) Gag variants into 293T cells. As controls, constructs containing wt Gag in combination with Pol with enzymatically inactive reverse transcriptase (iRT) or inactive integrase (iIN) domain, as well as constructs harboring translationally inactivated ORFs for Gag and Pol (ΔGag/Pol) or Env (ΔEnv) were used. The mock control (mock) included cells transfected with pUC19 alone. (A) Representative Western blot analysis of viral particles (virus) purified from 293T cell culture supernatant by ultracentrifugation through 20% sucrose and 293T cell lysates (cell). PFV proteins were detected using polyclonal antibodies specific for PFV Gag (α-Gag) or PFV Env LP (α-LP), a mixture of hybridoma supernatants specific for PFV Pol PR/RT and IN (α-PR/RT + α-IN), or a commercial monoclonal antibody specific for GAPDH (α-GAPDH). Serial dilutions of the wt samples (wt; lanes 6–9) were quantified to determine their relative protein contents compared to other samples. The identity of the individual proteins detected is indicated on the right. (B) Viral particle release was determined by quantitative Western blot analysis of viral particle lysates. Mean values and standard deviations (n = 3) are shown as relative values compared to the wt control and normalized for cellular expression levels. (C) Quantification of PFV vgRNA in virus producing cells (vgRNA cell) and released particles (vgRNA virus) and particle-associated vgDNA (vgDNA virus). Mean values and standard deviation (n = 3–8) are shown as relative values compared to the wt control. Cellular values were normalized to GAPDH levels, viral particle values were normalized for Gag content.

In order to understand the underlying causes for the replication defects of PFV particles with abolished Gag-PLK interaction, we characterized various features of the mutant viruses. We first harvested lysates of virus-producing cells after transient transfection of either four-component PFV vector system or full-length proviral PFV constructs into 293T cells. We also studied purified viral particles released into the supernatant to examine viral protein expression, particle release, viral genomic RNA (vgRNA) packaging and intra-particle reverse transcription (Fig 6; S6 Fig). None of the PFV Gag STP motif mutations affected the efficiency of virus-production, as implicated by analysis of viral protein and nucleic acid content. Particle release efficiency, viral protein composition and processing of these mutants were comparable to wt, as were their vgRNA and vgDNA contents in the context of both viral production systems (Fig 6; S6 Fig). Therefore, it can be concluded that the introduction of STP motif mutations into PFV Gag, which prevents interaction with cellular PLK proteins, does not have an effect on virus production, release, vgRNA packaging or intra-particle reverse transcription activity.

### Short-term pre-treatment of PFV wt infected cells with PLK inhibitor mimics PFV Gag iSTP/pmSTP mutant particles phenotype

The observed infectivity defects of PFV Gag STP motif mutant viruses pointed to an essential function of Gag-hPLK interactions at an early stage in viral replication. The results suggest that phosphorylation of a viral and/or cellular target, after binding of cellular PLKs to the phosphorylated Gag S-pT-P binding site, may be beneficial for PFV infection. To test this hypothesis we asked how enzymatic inhibition of hPLK1 might influence virus infectivity.

**Fig 7:**
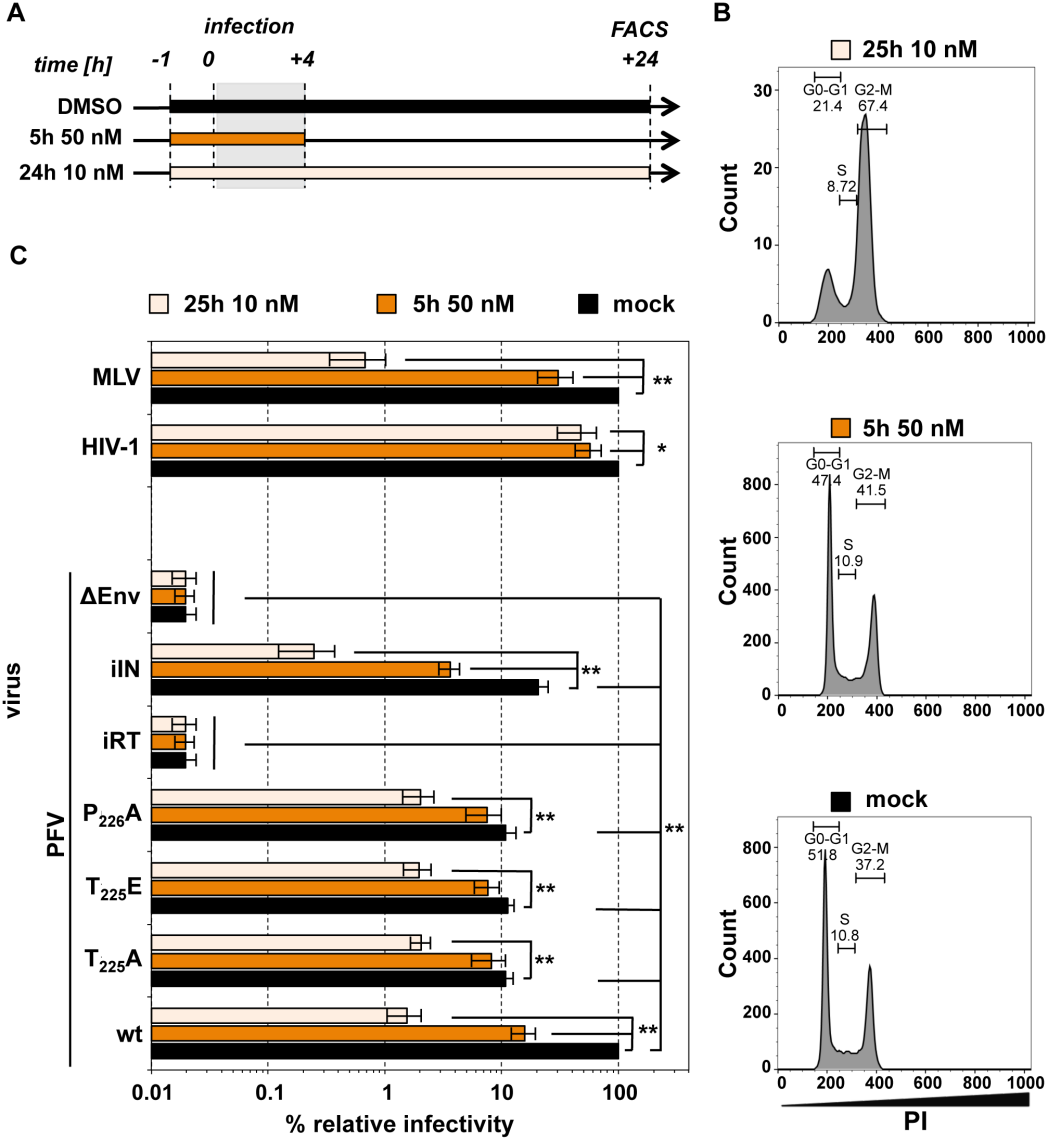
Effect of enzymatic PLK inhibition on the titers of PFV, HIV-1 and MLV virions. (A) Experimental outline. HT1080 target cells were infected with serial dilutions of the individual virus supernatants as indicated in the presence of the vehicle control (DMSO) or one of two BI-2536 concentrations. (B) Cell cycle profiles of mock infected cell populations of the three experimental groups determined by propidium iodide staining 25 h from the start of treatment (with either DMSO or BI-2536). (C) Virus infectivity was determined 24 h post-infection by flow cytometry analysis of infected target cell populations. The values obtained using wt variants of PFV, HIV-1, or MLV supernatants in combination with vehicle control treatment were arbitrarily set to 100%. Absolute titers of wt supernatants ranged between 5.0 x 10^5^ and 8.0 x 10^5^ (PFV), 1.9 x 10^6^ and 3.9 x 10^6^ (HIV), and 1.5 x 10^7^ and 2.5 x 10^7^ eGFP ffu/ml (MLV). Relative means and standard deviations from three independent experiments are shown. Differences between means of the respective wt viruses in combination with vehicle control and the individual mutants or treatment regimen with BI-2536 were analyzed by Welch’s t test (*, p<0.05; **, p<0.01).

For inhibition of cellular PLKs, in particular PLK1, during FV entry, HT1080 target cells were incubated with the well characterized PLK inhibitor BI-2536 [43] in two different regimens as outlined in Fig 7A. Because BI-2536 treatment is known to arrest cells in prometaphase in a dose and time dependent manner, we used propidium-iodide (PI) staining to determine the cell cycle status 24 h post-infection, at the time when productive infection was quantified by flow cytometry (Fig 7B). Short-term treatment of HT1080 target cells with 50 nM BI-2536 for 5 h (1 h prior to and 4 h during virus incubation) did not alter the cell cycle profile at 24 h post-infection as compared to the mock-treated population. This indicates that 50 nM BI-2536 treatment yields a largely cycling cell population and that short-term hPLK1 inhibition did not result in cell cycle arrest. In contrast, the target cell cycle profile was dramatically changed when 10 nM BI-2536 was applied for the entire 25 h examination period (long-term treatment). The target cell population was characterized by an altered cell cycle profile with highly increased G2-M population, indicating that hPLK1 inhibition by this inhibitor regimen led to a potent cell cycle arrest.

The influence of PLK inhibition by BI-2536 treatment revealed interesting, differential phenotypes for Gag wt and Gag STP mutant containing FVs (Fig 7C). When HT1080 target cells were treated with 50 nM inhibitor for only 5 h (short-term) the infectivity of wt PFV was reduced by 85% in comparison to mock treatment (Fig 7C, orange bars). In contrast, the infectivity of STP motif mutant particles (T225A, T225E, P226A) were only reduced by 25 to 30% in comparison to respective mock treated samples. Thus, the short-term inhibitor treatment reduced the infectivity of wt virus nearly to the level of Gag STP mutant viruses. A more prominent inhibitory effect was observed for the long-term 10 nM BI-2536 treatment regimen (Fig 7C, magenta bars). The infectivity of wt virus was again reduced to a much greater extent (65-fold) than Gag STP mutant viruses (5–6 fold) in comparison to the respective mock treatment controls, resulting in similar levels of residual infectivity. Taken together the data demonstrate that short-term PLK1 inhibition during entry of wt PFV, which does not induce a cell cycle arrest, can largely mimic the infectivity defect of virus mutants with Gag proteins unable to interact with cellular PLKs.

The effect of BI-2536 on viral infectivity was also examined for murine leukemia virus (MLV) and HIV-1 (HIV-1) vectors as representatives of retroviral genera that are dependent or independent of target cell mitosis for productive infection, respectively (Fig 7C). Both types of vectors were pseudotyped with PFV Env and harbored an identical reporter gene as transmitted by FV vector particles. Whereas HIV-1 infectivity was only weakly (2-fold) diminished under both treatment conditions, MLV infectivity was moderately inhibited (3-fold) by short-term PLK inhibition and very strongly reduced (150-fold) by long-term BI-2536 treatment. Hence, the data indicate that hPLK1 inhibition effects are not PFV-specific, which may encourage further insight into PLK proteins as a key link between retroviral infection and cell cycle progression.

### PFV Gag iSTP mutations do not affect virus target cell attachment, virus uptake or Gag protein stability

**Fig 8:**
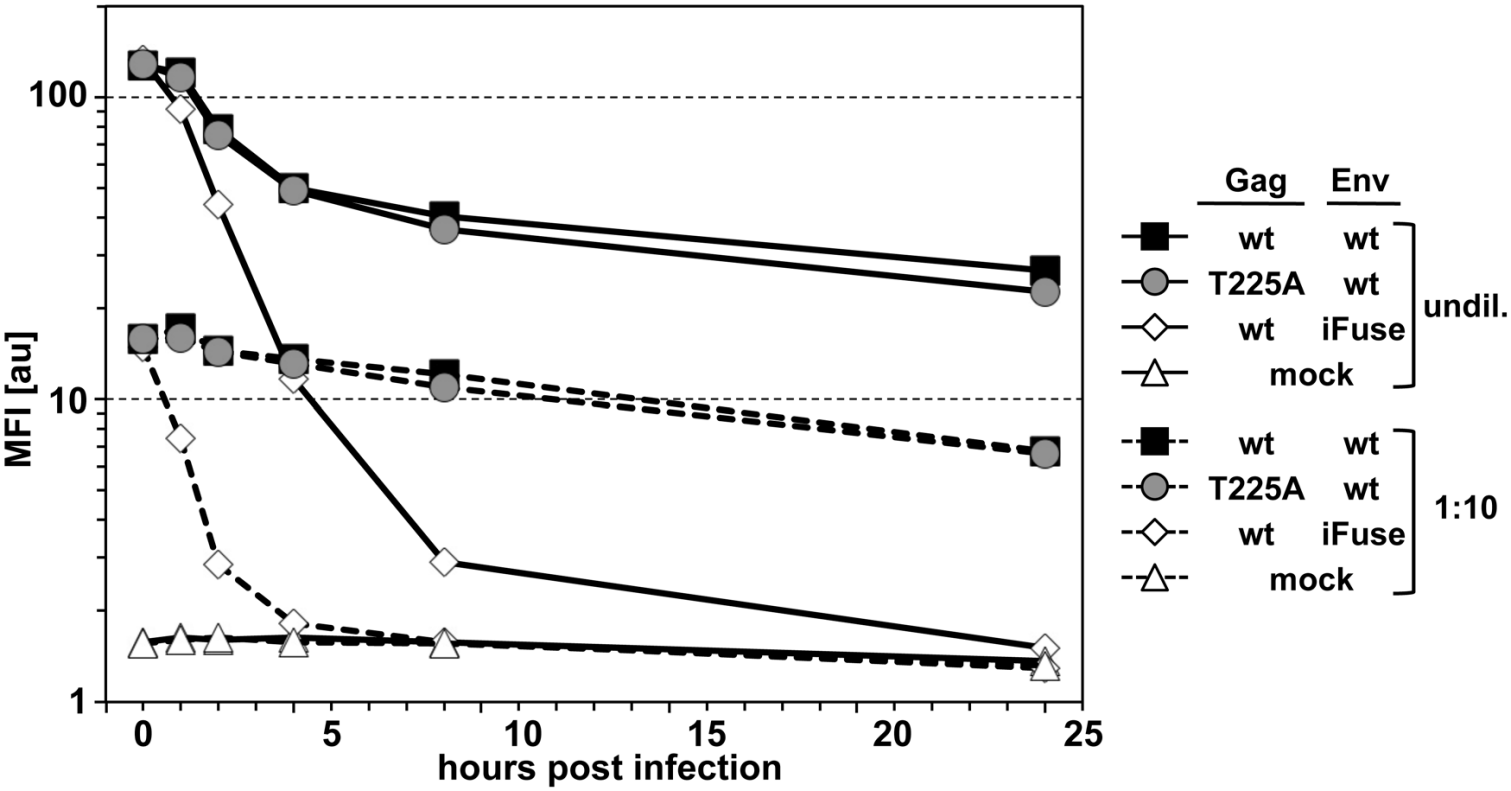
Analysis of wt and mutant PFV attachment, uptake and Gag protein stability. Replication-deficient PFV supernatants were harvested after transient transfection of the PFV four-component vector system, containing the eGFP-tagged Gag p68 wt (wt) or T225A (T225A) variants in combination with wt (wt) or fusion-incompetent Env (iFuse) into 293T cells. HT1080 cells were synchronously infected with undiluted (undil.) and ten-fold diluted (1:10) PFV supernatants and eGFP MFI values were measured by flow cytometry at indicated time points until 24 h post-transduction. Representative data shown are from one out of three independent experiments.

The reduced infectivity observed for PLK-interaction-deficient PFV or upon drug-mediated inhibition of PLKs during infection with wt PFV can result from defects at different steps during PFV entry and uptake into target cells. This includes early steps such as attachment, membrane fusion, capsid stability or later steps such as stability of the PIC or its tethering to host cell chromatin as well as vgDNA integration. To further narrow down which step might be affected we examined the uptake dynamics of wt and Gag T225A mutant virions containing fluorescently-tagged Gag proteins in synchronously infected HT1080 cells (Fig 8). At different time points after initiating virus uptake the amount of cell-associated, fluorescently labeled viral Gag protein was quantified by flow cytometric determination of the mean fluorescent intensity (MFI) of the whole cell population. As non-infectious control GFP-tagged Gag wt containing particles harboring a fusion-deficient PFV Env glycoprotein (iFuse) were used. All types of virions similarly attached to target cells by spinoculation at low temperature (Fig 8). Fusion-deficient virions (wt iFuse) were rapidly internalized upon raising the temperature and the Gag-GFP signal declined to background levels within 4 to 24 h depending on the amount of virus used for infection. This most probably is the consequence of rapid degradation of non-fused particles by endocytic uptake and targeting to lysosomes [44, 45]. In contrast, Gag wt (wt wt) and Gag T225A (T225A wt) samples showed as early as 1 h post-infection significantly higher MFI signals than the samples of fusion-deficient virions (wt iFuse), and this difference further increased over time. In line with previous studies [44, 45], this indicates rapid release of capsids into the cytoplasm for virions with fusion-competent, wt glycoprotein. Cytoplasmic PFV capsids are known to be quite stable and transported along microtubules to the MTOC of the host cell, where they await host cell entry into mitosis for progression towards virus genome integration into cellular chromatin [18]. In the examined time period of up to 24 h post-infection, no significant differences in the Gag-GFP MFI profiles of wt and Gag T225A mutant particles were observed, independent of the amount of virus initially attached to HT1080 cells.

Taken together the analysis indicates similar attachment, fusion and uptake dynamics as well as Gag protein stability for wt and Gag T225A virions. This suggests a defect at a step post-disassembly of PFV capsids as responsible for the observed infectivity phenotype of iSTP mutant PFV virions.

### PFV mutants deficient in PLK interaction display delayed and decreased integration efficiency

**Fig 9:**
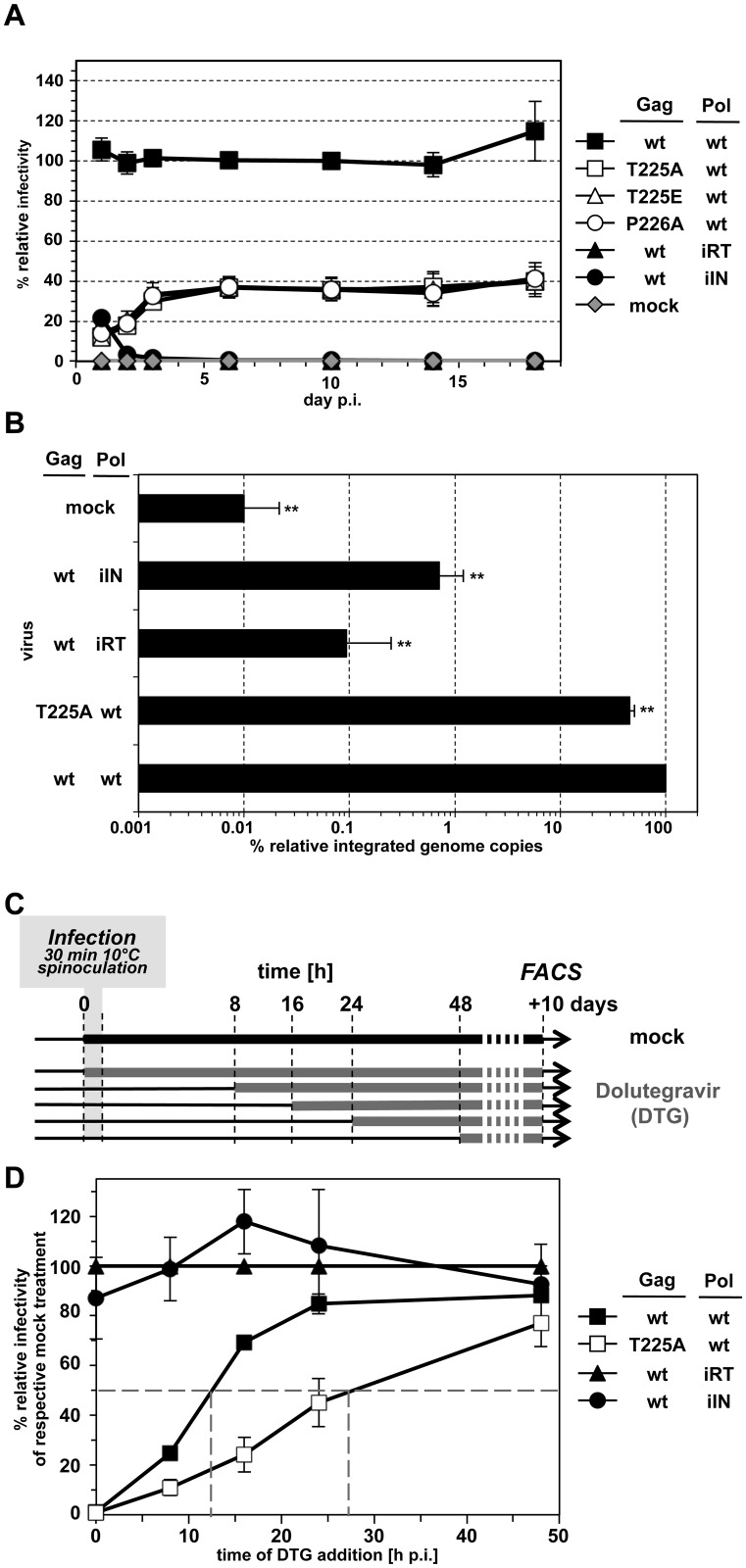
Integration efficiency and dynamics of PFV wt and STP mutant virions. (A) Virus infectivity was determined at different time points post-infection by flow cytometry analysis of infected target cell populations as indicated. The values obtained using wt PFV Gag expression plasmids were arbitrarily set to 100%. Relative means and standard deviations normalized for Gag content (except mock) from two independent experiments in duplicates are shown. Absolute titers of wt supernatants ranged between 5.7 x 10^4^ and 9.3 x 10^4^ (1 day p.i.), 8.6 x 10^5^ and 1.5 x 10^6^ eGFP ffu/ml (18 day p.i.). (B) Comparison of wt and STP mutant integration efficiencies. HT1080 target cells were infected with wt, T225A, iRT, iIN and ΔEnv (mock) supernatants. Ten days post-infection, genomic DNA was isolated from target cells and provirus numbers integrated into the host cell genome were quantified by Alu-qPCR and normalized to ß-actin copy numbers. The values obtained using wt PFV Gag expression plasmids were arbitrarily set to 100%. Relative means and standard deviations from three independent experiments in duplicates are shown. (C) Integration dynamics of wt and STP mutant viruses analyzed by inhibition of viral infectivity by dolutegravir addition at different time points post-infection and maintenance until flow cytometric determination of viral titers 10 days post-infection. (D) The values obtained for each respective virus type without DTG were arbitrarily set to 100%. Relative means and standard deviations from three independent experiments are shown.

Closer examination of reporter gene expression in target cells transduced with wt or Gag STP motif mutant PFV particles over time revealed marked differences in the severity of STP mutant infectivity defect (Fig 9A). The largest differences in STP motif mutant vector titers, up to 10-fold reduced compared to wt, were observed if the number of reporter gene expressing target cells used for virus titer calculation were quantified 1 day post-transduction in single-round infection experiments (Fig 9A). Up to 3 days post-transduction this difference constantly declined until reaching a plateau of 3-fold reduced infectivity in comparison to wt. This phenotype of a decrease in viral infectivity and the time-dependent quantitative differences may suggest a combination of delayed and reduced integration potential of iSTP mutant PFV particles in comparison to wt virus.

To gain support for this hypothesis, we extracted genomic DNA from HT1080 cells 10 days post-infection with different virus supernatants and determined integrated PFV genome copy numbers by quantitative Alu PCR (Fig 9B). The number of integrated vector genomes of cells infected with PFV Gag T225A containing virus supernatants was reduced to 45% of wt and correlated well with their reduced infectivity. Thus, the reduced viral titer of PFV Gag T225A containing particles determined by the reporter gene transfer assay and flow cytometric analysis late after transduction is mainly the consequence of a reduced number of proviral integration events.

Attenuated integration would suffice to explain the defects in mutant virus titers, but not the delay in the number of viral DNA-derived reporter gene expressing cells observed for the STP mutants during the first three days after infection. To further characterize the iSTP/pmSTP integration dynamics, we made use of a well-characterized HIV-1 integrase inhibitor, dolutegravir (DTG) [46], previously shown to also be active against PFV integrase [47]. The rationale behind this set of experiments was that if wt and iSTP mutant viruses integrated their genomes at different dynamic rates during the initial 72 h post-infection, then they should be differentially sensitive to DTG treatment over time. Following this notion, we synchronously infected HT1080 cells with wt (wt wt) or T225A mutant (T225A wt) PFV and mock solvent (DMSO) or 2 μM DTG at different time points post-infection and maintained the drug until flow cytometry analysis was performed at day 10 post-infection (Fig 9C). As control, cells were infected with supernatants harboring viral particles containing wt PFV Gag in combination with Pol variants with either enzymatic inactive reverse transcriptase (wt iRT) or integrase (wt iIN) domains. As expected, DTG addition to samples infected with the latter viruses (wt iRT, wt iIN) did not influence their infectivity in comparison to respective mock treated controls (Fig 9D). In contrast, the infectivity of both Gag wt (wt wt) and Gag T225A (T225A wt) containing viral particles was efficiently inhibited (to ~1% of respective mock treated samples) when DTG was added at the time of virus addition (0 h time point) (Fig 9D). Strikingly, the infectivity of both types of viruses was differently affected when DTG was added at later time points. Gag wt (wt wt) containing particles became resistant to DTG-mediated inhibition quickly and acquired 50% drug resistance at ~12 h post-infection whereas Gag T225A (T225A wt) containing particles reached this value much later at ~27 h post-infection. These results indicate that approximately half of wt PFV proviruses establish within 12 hours post-infection and confirm that Gag STP mutations cause a significant delay in PFV integration.

### Increased preference for heterochromatin targeting by Gag iSTP mutant viruses

In sharp contrast to orthoretroviruses such as HIV-1 and MLV that predominantly integrate within or in the vicinity of actively expressed genes, PFV disfavors integration into transcriptionally active chromatin and within transcription units [48–50]. To test if the delayed integration dynamics of PFV Gag iSTP mutants influences their integration site preferences, we interrogated integration site distributions of PFV vectors produced using S224A and T225A mutants of the Gag packaging vector (pcoPG4). The integration sites produced by infection of HT1080 cells were amplified using ligation-mediated (LM)-PCR as described [48]. Pools of amplified fragments containing junctions of downstream (U5) ends of vgDNA and chromosomal DNA were sequenced using the Illumina MiSeq platform, and precise positions of integration sites were mapped to the hg19 version of the human genome. To assess data reproducibility, PFV wt integration sites were determined from three independent viral infection experiments whereas mutant sites were collected from two independent sets of infections. In total, 185,481 WT, 44,918 S224A, and 40,455 T225A sites were mapped across experiments (Table 1). A previously described dataset of ~2.2 million unique integration sites obtained using recombinant PFV intasomes and isolated human genomic DNA *in vitro* was used as a reference [48]. Such a reference incorporates the innate preferences of PFV integrase for chromosomal DNA sequence, whatever they may be, as well as variations of PCR amplification efficiency depending on local G/C content or repeat density.

**Table 1:** PFV Integration Site Distribution in Individual Experimental Samples.

*Sample*	*Unique Sites*	*% in RefSeq*	*% +/- 2*.*5kb Lamin*	*Gene Density/1Mb*
**WT-1** ^a^	31275	29.8	68.5	6.3
**WT-2**	100234	32.2	65.6	6.6
**WT-3**	53972	32.1	65.8	6.7
**S224A-1** ^a^	37293	29.7	73.4	5.3
**S224A-2** ^b^	7625	29.7	72.8	5.4
**T225A-1** ^a^	29623	30.0	73.7	5.3
**T225A-2** ^b^	10830	30.5	72.4	5.6
**HIV-1**	335725	74.6	15.8	19.9
**in vitro**	2212496	47.5	39.8	10.3

^a^ Infections performed on the same day.

^b^ Infections performed on the same day.

**Fig 10:**
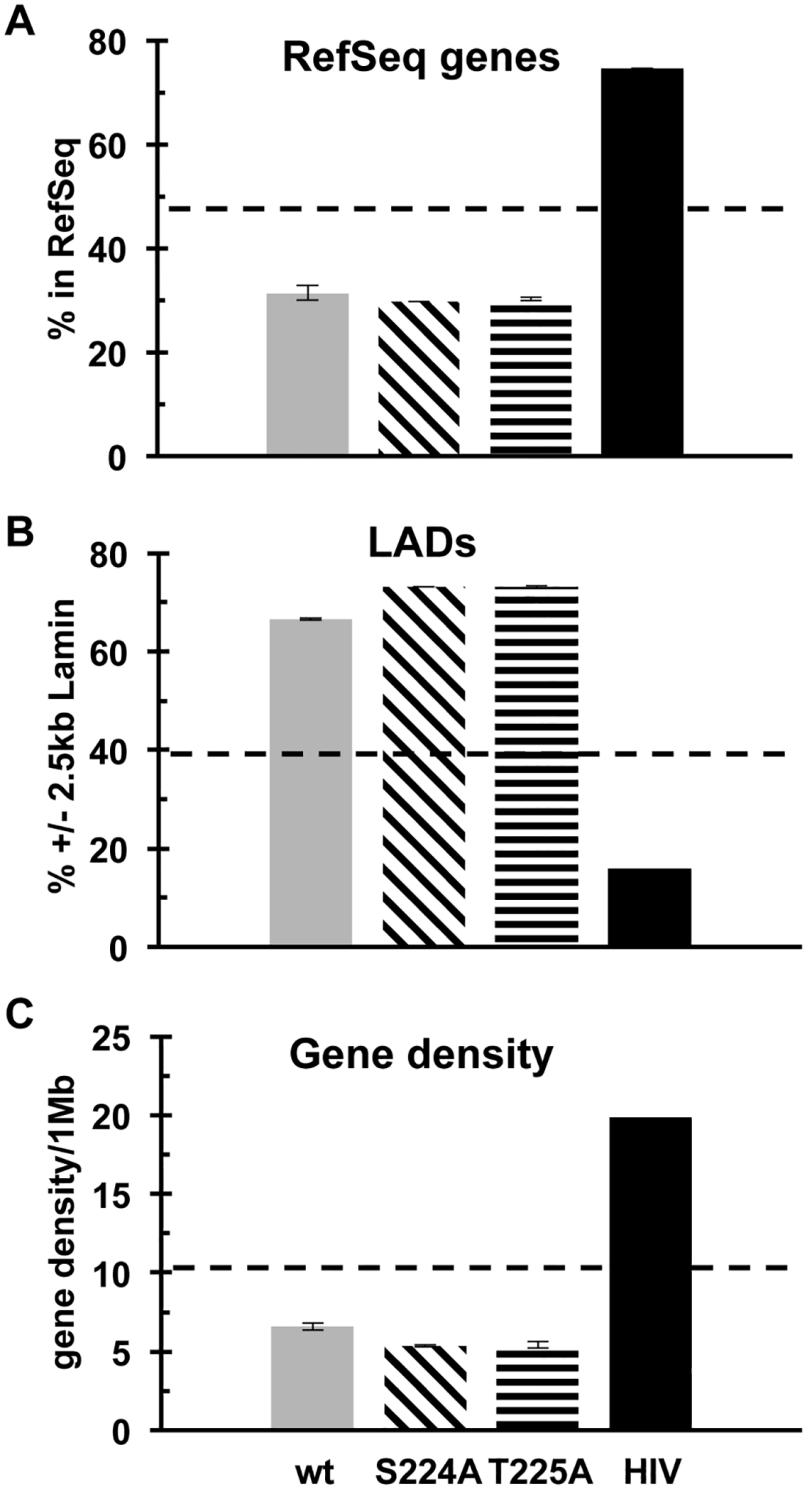
Integration site profiles of WT and Gag iSTP mutant viruses. (A) Percent of WT (gray bar), S224A (backslash), and T225A (horizontal slash) integrations within RefSeq genes. An independent HIV-1 dataset (black) [85] was included for comparison. (B) Percent of integrations within lamina-associated domains (LADs). (C) Average gene density in 1 Mb regions surrounding the integration sites. The data from three wt and two S224A and T225A integration site libraries (Table 1) were combined, with error bars indicating the resulting standard deviation. Dotted, horizontal lines represent the percent of integrations from the *in vitro* integration dataset. Please refer to S7 Fig for statistical analyses.

In agreement with previous observations, wt PFV targeted genes 31.4% of the time across datasets, which was highly statistically significant from the matched *in vitro* frequency of 47.5% (Fig 10A; S7 Fig). Due to the relatively large numbers of analyzed integration sites, the levels at which genes were targeted yielded statistical significance across some wt datasets. Given this, gene targeting by the iSTP Gag mutants was largely indistinguishable from the wt. This is especially evident by comparing the WT-1 and mutant datasets, which were derived from viral infections performed on the same day (P values of 0.87 for S224A-1 and 0.58 for T225A-1; S7 Fig). By contrast, the targeting of heterochromatin, which is naturally preferred by PFV, was reproducibly hyper-targeted by the Gag mutants. Thus, mutant viral targeting of lamina-associated domains (LADs) was significantly enhanced (Fig 10B; P values 4.1 x 10^−46^ for matched S224A and 8.4 x 10^−47^ for matched T225A infections–S7 Fig) whereas the targeting of gene dense regions of chromosomes was significantly decreased (Fig 10C; matched S224A and T225A P values of 1.2 x 10^−55^ and 1.3 x 10^−61^, respectively).

## Discussion

The timing of integration of some retroviruses including MLV and FVs has long been recognized to coincide with mitosis [reviewed in 51], but until now no explanation on how this process is coordinated has been offered. Since host cell division is a major limiting factor for the broad use of FV (and some other retroviral) vectors for transduction of resting target tissues, knowledge of the cause of this limitation may help to improve the existing gene therapy vector systems [8].

In this study, we identified members of the hPLK family as functional co-factors of PFV replication. By using both yeast and mammalian cell based systems, we show that PFV Gag interacted with PLK1-3 in yeast and recruited PLK1-2 to condensed human mitotic chromatin. The failure of FL PFV Gag to interact with FL PLK1 but not with FL PLK2 or FL PLK3 in Y2H assays may indicate a differential structural requirement for Gag to interact with FL PLK1 compared to FL PLK2 or FL PLK3. FL PLK1 only interacted with C-terminally truncated PFV Gag baits lacking at least 104 aa encompassing half of the GR-rich region, which is known to be functionally relevant for capsid morphology and other functions. Therefore it may be speculated that a differential oligomeric structure of PFV Gag mutants may be responsible for the differential interaction phenotype with individual FL PLKs in yeast. The interactions of PFV Gag with PLKs in yeast and mammalian cells depended on a central domain in PFV Gag containing a consensus S-(T/S)-P PLK binding site [29], which is evolutionary conserved in all primate FVs. Similar as reported for STP binding sites of other PLK substrates [33, 52, 53], phosphomimetic mutations of the central STP motif residue did not restore interaction of PFV Gag with the examined PLK proteins. This study for the first time identified this sequence in PFV Gag as a motif important for interaction with cellular PLKs and its conservation across primate FV Gag species points to a potentially general role of this interaction in primate FV replication. Interestingly, Gag proteins of other retroviruses, such as MLV, Rous sarcoma virus, gibbon ape leukemia virus or human T-lymphotropic virus 4 harbor S-(T/S)-P peptide motifs that upon phosphorylation may function as PLK binding sites. In line with this, the MLV vector used in this study showed a similar response profile towards target cell PLK inhibitor treatment as PFV.

PFV Gag of released virions was phosphorylated to a significant extent at T225, whereas Gag phosphorylated at threonine residues was not detectable in immunoprecipitates from virus producing 293T cells. This may be simply a consequence of overpowering the cells by transient overexpression of PFV Gag. Alternatively it may suggest that only a minor proportion of cellular PFV Gag is modified by threonine phosphorylation, potentially in a cell-cycle dependent manner, and that Gag phosphorylated at T225 and other residues (as detected by the pT-P antibody staining) is enriched in secreted virions. It will be interesting to determine whether only Gag in preassembled capsids or also monomeric Gag harbor phosphorylated PLK binding sites and if Gag phosphorylation is dependent on the cell cycle status of the expressing cell. While the phenomenon of the apparent enrichment of phosphorylated Gag in secreted virions awaits mechanistic characterization, it is plausible to speculate that Gag phosphorylation, not only at T225 but also other residues, may influence Gag protein oligomerization and functional capsid formation as well as their trafficking and/or their interaction with the cytoplasmic domain of the Env LP subunit, which is essential for PFV budding [54].

It has been shown previously that PFV Gag is phosphorylated on multiple serine residues [55], but no kinase(s) responsible for this process had been identified. Therefore, it will be important to decipher the cellular kinase(s) that mediate Gag phosphorylation at T225 and/or other serine/threonine residues. Likely candidates are Cdk1, Cdk2 or PLKs themselves. One speculation concerning the biological function of FV Gag phosphorylation could come from the analogy of their replication strategy to hepadnaviruses. Serine phosphorylation of the HBV core protein has important functions in reverse transcription and virion morphogenesis [56–59]. However, the analysis of consequences of PFV Gag T225 phosphorylation suggests rather a role during the early events of PFV replication than for virus morphogenesis processes as observed for HBV.

On the PLK side, the assays using rPLK2 as a representative revealed that in addition to a functional PBD, kinase activity is required for the observed protein-protein interactions. This is reminiscent of reports for some other PLK2 substrates in neuronal tissues, for which the PLK2 ATP-binding pocket in addition to the integrity of the STP phosphorylation site are important for driving interaction between these two partners [23, 60]. It was suggested that phosphorylation and ATP binding induce structural and conformational changes in PLK2 and its binding partner to facilitate their interaction, which may also be the case for PFV Gag-PLK interactions. Alternatively, potential PFV Gag phosphorylation by PLKs in cis, at yet to be identified sites, or the trans phosphorylation of other currently unknown components complexed with Gag, may stabilize the observed interactions and be crucial for the PLK-mediated role in PFV replication. However, it has to be determined whether similar requirements in respect to a functional KD as observed for rPLK2 are essential for the interaction of PLK1 and 3 with PFV Gag as well.

The functional analysis of the FV Gag—PLK interaction for viral replication suggests a significant role in promoting timely and efficient PFV vgDNA delivery into the nucleus, as revealed by a detailed comparison of wt virions to those containing Gag with STP mutations. While the single amino acid exchanges of each of the residues in the Gag PLK recognition and binding motif did not alter virus particle production, vgRNA encapsidation or reverse transcription, they resulted in diminished infectivity of Gag STP mutant particles, underlined by the delayed and decreased vgDNA integration efficiency compared to wt. The particle-associated Gag STP mutants were likely unable to interact with cellular PLK proteins after entry into target cells, due to the deficient STP motif phosphorylation and the resulting lack of recognition by the PLKs. Along this line, future studies should address the details and mechanistic insights into PFV Gag phosphorylation as mentioned before and should characterize the spatial-temporal requirements of the PFV capsid interaction with one or more of the mammalian kinase families.

By dissecting various individual steps in PFV uptake and entry, we determined that the iSTP mutant viruses display wt-like attachment, entry and Gag protein stability characteristics, but that their vgDNA genome integration is delayed and reduced compared to wt virions. The successful diminishment of wt PFV infectivity to the level of the STP mutants by drug-mediated PLK enzymatic inhibition in infected cells suggests that the reduced STP mutant infectivity is not induced by the lack of effective PFV Gag interaction with host cell PLK proteins per se. The abolished association of PLK enzymatic activity with FV capsids appears to be responsible for the STP mutant virus phenotype, because the observed effects of the PLK inhibitor on viral infectivity were dependent on the presence of wt Gag protein in PFV virions, although we currently cannot formally exclude an inhibition of the Gag—PLK interaction by the enzymatic inhibitor. Furthermore, the similar reduction of iIN mutant virus infectivity upon drug-mediated inhibition of PLK activity suggests that the replication step that the PLK-Gag interaction likely potentiates is nuclear import and/or tethering to host chromatin. Importantly, these experiments revealed that MLV, but not HIV-1, virions were sensitive to PLK inhibition in a PFV wt-like manner. Interestingly, the MLV Gag contains a unique STP motif at amino acid positions 123–125 in the MA subunit, the significance of which for virus replication to our knowledge has not yet been characterized. Thus, we suspect that the ability for similar Gag domains to confer interaction with one or more cellular PLKs may contribute to the mitosis dependence for PFV and MLV infection.

As BI-2536 has the highest specificity for PLK1, but also inhibits PLK2 and PLK3, it is currently unclear whether interactions with a single or multiple PLKs are involved in PFV replication. Although PLK1 appears the most likely candidate, the exact role of PLK2 (and possibly also PLK3) needs to be addressed in the course of further studies. Furthermore, it will be important to resolve the spatio-temporal involvement of different PLKs in PFV replication (e.g. by applying live cell imaging studies of PFV-infected cells) to better understand the mechanistic details of these interactions. Furthermore, since mutant PFV unable to interact with cellular PLKs retain a residual infectivity it may be speculated that likely currently unknown interactions of the virus and/or capsid with other cellular components of the cell cycle regulatory machinery exist, which provide functions redundant to or compensatory for the Gag—PLK interaction characterized in this study.

Finally, we observed that PFV Gag STP mutant viruses achieved only 45% of wt levels of proviral integrations, suggesting that 55% of vgDNA copies taken up are lost. Furthermore, statistically significant alterations in integration site profile, with a shift towards an even higher preference for heterochromatic regions than wt PFV, was detected for mutant viruses. Together these two features may account for the roughly 3-fold reduced infectivity of PLK-binding deficient viruses. The underlying mechanism responsible for this phenotype are currently unknown, but may be due to untimed mutant capsid disassembly and/or faster degradation of mutant PICs in host cells. Alternatively cellular PLKs may represent the first tethering- and/or integration-promoting host cell factors involved in PFV replication. Perhaps the PFV Gag-PLK interaction and subsequent phosphorylation of Gag itself or other proteins in PFV PICs influences their integration site selection profile. Even though it is uncertain whether PLK protein(s) may mediate roles similar to those of LEDGF/p75 and BET proteins in HIV and MLV integration, respectively [reviewed in 61], the presented data offer initial insight into how PFV may coordinate its timely genome nuclear delivery with host cell mitosis.

Analysis of the amino acid sequence of other PFV structural proteins revealed that PFV Pol also harbors two potential STP motif consensus binding sites, which are located in the integrase domain. However, follow up analyses, which included Y2H using Pol or its processed integrase subunit as baits as well as functional analysis of single-round vector particles with point mutations in single or both Pol STP binding motifs, failed to indicate a biologically relevant interaction between Pol/integrase and PLK or a role in PFV infectivity. This highlights that it is the interaction of cellular PLKs with PFV Gag and not Pol that are important for timely and efficient proviral integration. The capsid protein of HIV-1 has similarly been shown to play a significant role in integration through its cellular binding partner CPSF6 [62]. Although our data indicate that the two STP motifs in PFV Pol appear not to be of functional relevance for viral infectivity, they do not formally exclude the possibility that cellular PLKs in complex with PFV Gag mediate phosphorylation of PFV integrase residues upon virus entry and thereby influence integrase function. Which functionally relevant residues in integrase thereby might be posttranslationally modified needs to be determined, however, our data suggest, that it is not T961 or T1058 within the integrase STP motifs.

## Materials and Methods

### Cells and culture conditions

The human embryonic kidney cell line 293T (ATCC CRL-1573) [63], the human epithelium HeLa cell line (ATCC CCL-2) [64], the human primary fibroblast MRC-5 cell line (ATCC CCL171), immortalized mouse primary embryonic fibroblasts (obtained from M. Trilling, Essen, Germany) and the human fibrosarcoma cell line HT1080 (ATCC CCL-121) [65] as well as the clonal variant HT1080 PLNE thereof containing a PFV LTR driven EGFP reporter gene expression cassette [66] were cultivated in Dulbecco’s modified Eagle’s medium (DMEM) supplemented with 10% heat-inactivated fetal calf serum and antibiotics. HeLa cells used for confocal laser scanning microscopy were cultivated in phenol red free media. For hPLK1 and PFV integrase inhibition experiments, BI-2536 (Selleckchem) or Dolutegravir (Selleckchem) dissolved in DMSO was used at final concentrations as indicated.

### Recombinant plasmid DNAs

A four-component PFV vector system, consisting of the expression-optimized packaging constructs pcoPG4 (PFV Gag), pcoPE (PFV Env), pcoPP (Pol), and the enhanced green fluorescent protein (eGFP)-expressing PFV transfer vectors pMD9 or puc2MD9, has been described previously [67–69]. In some experiments previously described variants of the PFV Pol packaging construct, containing ORFs with catalytically inactive reverse transcriptase (pcoPP2, Pol iRT, YVDD_312–315_GAAA mutation) or catalytically inactive integrase (pcoPP3, Pol iIN, D_936_A mutation), were used [66, 67].

All PFV Gag packaging constructs used in this study are based on the parental pcoPG4 vector or its C-terminal mCherry and eGFP tagged variant pcoPG4 CCh and pcoPG4 CEG [68]. The PFV Gag packaging constructs encoding mutant Gag protein with alterations in a putative PLK STP binding motif (pcoPG4 S224A, pcoPG4 T225A, pcoPG4 T225D, pcoPG4 T225E, pcoPG4 P226A) generated for and used in this study are depicted in Fig 1D.

All PFV Pol packaging constructs used in this study are based on the parental pcoPP vector [67]. PFV Pol packaging constructs encoding mutant Pol protein with alterations in the two putative PLK STP binding motifs, either individually (pcoPP6 [S960A], pcoPP7 [S1057A]) or in combination (pcoPP8 [S960A+S1057A]), were generated by recombinant PCR techniques and used in this study.

The CMV-driven proviral expression vector pczHSRV2 (wt) and its variants pczHSRV2 M69 (iRT), expressing a Pol protein with enzymatically inactive RT domain (YVDD_312-315_GAAA mutation), and pczHSRV2 M73 (iIN), with enzymatically inactive IN domain were described previously [70, 71]. For this study the variants with altered putative PLK binding motif, pczHSRV2 S224A (S224A), pczHSRV2 T225A (T225A), pczHSRV2 T225E (T225E), pczHSRV2 P226A (P226A), were generated (Fig 1E).

Lentiviral PFV Env pseudotypes were generated using the mutant PFV Env packaging vector pcoPE01, the HIV-1 Gag-Pol expressing packaging vector pCD/NL-BH and the HIV-1 based transfer vector p6NST60 [44, 72, 73]. MLV PFV Env pseudotypes were produced using pcoPE01, the MLV Gag-Pol expressing packaging vector pHIT60 and the MLV based transfer vector pczCFG2 fEGFPf [44, 74].

Y2H expression constructs containing the GAL4 activation domain (GA) with C-terminal (pCGA) or N-terminal (pNGA and pNGA2) ORF fusions were based on pGADT7 (Clontech). Y2H constructs containing the GAL4 DNA-binding domain (GB) with C-terminal (pCGB) or N-terminal (pNGB) ORF fusions were based on pGBKT7 (Clontech) (Fig 1B). The respective Gag sequence for authentic, full-length wt Gag Y2H constructs was amplified from pcziPG4 [69] whereas constructs containing sequences of human expression-optimized Gag were based on pcoPG4 [68]. The Gag sequence for PFV Gag truncation Y2H constructs (Gag 1–621, Gag 1–594, Gag 1–544, Gag 1–482, Gag 1–450, Gag 1–350, Gag 1–310, Gag 1–297, Gag 1–295, Gag 1–212, Gag 1–129, Gag 130–648, Gag 156–648, Gag 183–648, Gag 230–648, Gag 130–295, Gag 183–295, and Gag 296–648) was amplified from the respective pcziPG4 truncation mutant expression constructs, which have been described previously [75]. Y2H PFV Pol expression constructs were generated inserting either the ORF of pcoPP1 (iPR) encoding a full-length Pol protein with enzymatically inactive PR domain or aa 752–1143 of pcoPP comprising the mature PFV Pol IN subunit into pCGA, pNGA, pCGB or pNGB expression vectors [66, 67]. Y2H pCGB and pCGA variants with full-length hTsg101 ORF were obtained from Wesley Sundquist and have been described previously [76].

CMV-driven mammalian expression constructs with ORFs encoding human PLK1 to PLK4 (hPLK1 to 4) were obtained from Rene Medema [77], and encoding wt rat PLK2 (rPLK2 wt) and point mutants thereof from Daniel Pak [60] (Fig 1D). ORFs encoding human (hPLK5) and mouse PLK5 (mPLK5) were purchased from Imagenes and subcloned into CMV-driven expression vectors. For some experiments N-terminally eGFP-tagged variants of hPLK1 to 3 (peGFP-PLK1 to 3) were used (Fig 1D). For Y2H interaction analysis the full-length ORFs of hPLK1 to 3, 5, mPLK5, rPLK2 and point mutants thereof, as well as the polo-box domains of hPLK1 to 4 were inserted C-terminal to the GAL4 activation domain (pCGA) into pGADT7 (Clontech) (Fig 1B).

All constructs were verified by sequencing analysis. Primer sequences and additional details are available upon request.

### Transfection and virus production

Cell culture supernatants containing recombinant viral particles were generated by transfection of the corresponding plasmids into 293T cells using polyethyleneimine (PEI) as described previously [37, 66]. For subsequent Western blot analysis the supernatant generated by transient transfection was harvested, passed through a 0.45-μm filter and centrifuged at 4°C and 25,000 rpm for 3 h in a SW32Ti rotor (Beckman) through a 20% sucrose cushion. The particulate material was resuspended in phosphate-buffered saline (PBS). For Western Blot analysis using phosphopeptide-specific antibodies viral particles were resuspended in TBS. Subsequently, half of the sample was digested for 1 h at 30°C with Lambda Phosphatase (NEB, P0753; 7.15 U/μl Lambda PP, 1% Triton X-100, 1 mM MnCl_2_, 1x Lambda Phosphatase buffer) whereas the other half was mock digested prior to addition of protein sample buffer.

### Infectivity analysis

Transduction efficiency of recombinant, eGFP-expressing PFV vector particles by fluorescence marker-gene transfer assay was analyzed at various time points post-transduction as described previously [44, 68, 78]. For synchronized infections a modified spinoculation protocol was used as described previously [44]. Virus particles generated by use of proviral expression plasmids were titrated on HT1080 PLNE cells harboring a Tas-inducible nuclear *egfp* ORF in their genome as described previously [66]. All transduction experiments were performed at least three times. In each independent experiment the values (eGFP focus forming units per ml, eGFP ffu/ml) obtained with the wt construct pcoPG4 and pczHSRV2, respectively, were arbitrarily set to 100% and values obtained with other constructs were normalized as a percentage of the wt values.

For evaluation of the effect of Dolutegravir (DTG) inhibition of PFV integrase enzyme activity on the infectivity of various PFV viruses HT1080 cells were plated at 2 x 10^4^ cells per well in 12-well plates 24 h prior to infection. Target cells were synchronously infected PFV Gag wt or Gag T225A mutant containing viral supernatants by spinoculation as described previously [44]. Briefly, target cells loaded with virus particles by incubation with 1 ml virus and centrifugation at 960x g and 10°C for 30 min. Subsequently, unbound virus was removed by a wash step and uptake and infection of virions attached to the cell surface initiated by shifting samples to 37°C in fresh growth medium. Two different amounts of PFV Gag wt or Gag T225A containing four-component vector system supernatants were used, which yielded in about 50% or 25% GFP positive cells, respectively, determined by flow cytometry analysis at 10 days post-infection in mock treated samples. As controls, cells were infected with supernatants harboring PFV Gag wt in combination with Pol variants with either enzymatic inactive RT (iRT) or IN (iIN) domains, at the highest amount of virus supernatant used. DTG stock (2 mM in DMSO) or an identical amount of solvent alone was added at different time points during infection as indicated in the experimental outline in Fig 9C to obtain a final concentration 2 μM DTG in the growth medium. Once added, DTG was replenished every second or third day until 10 days post-infection when the percentage of GFP expressing target cells of the individual samples was determined by flow cytometry analysis. These values were used to calculate viral titers of individual samples as described previously [78] and the effect of the time point of DTG addition on relative viral infectivity of the individual viruses plotted in respect to their respective mock treated control whose viral titers were arbitrarily set to 100%. Thereby a bias of the roughly 3-fold reduced infectivity of T225A Gag containing virions in comparison to wt was avoided.

### Virus uptake analysis

For synchronized infections using virions with GFP-tagged Gag proteins a modified spinoculation protocol was used as described previously [44]. Briefly the protocol involved preincubation of target cell (5 x 10^4^ cells/well plated in 12-well dishes one day in advance) at 10°C for 10 min. Subsequently the growth medium was replaced with 1 ml cold virus supernatant with the plates kept on cool pads. Next the viral supernatant containing tissue culture plates were centrifuged for 30 min at 960 x g at 10°C in a tissue culture centrifuge for loading of viral particles onto the target cells but preventing their endocytic uptake or glycoprotein-mediated membrane fusion. Uptake and infection were then initiated by replacing the viral supernatant containing all non-adsorbed vector particles with fresh warm growth medium and incubation at 37°C, 5% CO_2_ for the time periods indicated. Duplicate target cell wells plated were simultaneously transduced by spinoculation and samples were harvested for flow cytometry analysis consecutively at different time points. For analysis of the level of Gag-GFP protein cell association the mean fluorescent intensity (MFI) in the GFP channel of cultures transduced with different amounts viral supernatants (undiluted or 1:10 diluted cell-free 293T virus supernatant) was determined from all living cells as gated by their FSC/SSC profile.

### Western blot analysis

Cells from a single transfected 100-mm cell culture dish were lysed in detergent-containing buffer and the lysates were subsequently centrifuged through a QIAshredder column (QIAGEN). Protein samples from cellular lysates or purified particulate material were separated by SDS-PAGE on a 10% polyacrylamide gel and analyzed by immunoblotting as described previously [54]. Polyclonal rabbit antisera specific for PFV Gag [79] or the amino acids (aa) 1 to 86 of the PFV Env leader peptide (LP), [54] as well as hybridoma supernatants specific for PFV PR-RT (clone 15E10) or PFV IN (clone 3E11) [80], or a GAPDH-specific monoclonal antibody (G8795; Sigma), or a commercial phospho-specific mouse monoclonal antibody (9391, Cell Signaling) recognizing phospho-Thr-Pro peptides (pT-P) in a highly context-independent fashion, were employed. Furthermore, a custom-made (Abmart), affinity purified, phosphopeptide specific, rabbit polyclonal antiserum recognizing the phosphorylated PFV PLK STP binding site (PFV Gag S-pT-P) was generated by immunization with PRATS(pT)PGNIP peptides. After incubation with species-matched horseradish peroxidase (HRP)-conjugated secondary antibody, the blots were developed with Immobilon Western HRP substrate. The chemiluminescence signal was digitally recorded using a LAS-3000 (Fujifilm) imager and quantified using ImageGauge (Fujifilm).

### Mass spectrometric analysis

For mass spectrometric analysis (performed by the Mass Spectrometry Facility at the Max Planck Institute of Molecular Cell Biology and Genetics, Dresden) 200 ml cell culture supernatants of 293T cells transfected either with wild type constructs of the 4-component PFV vector system (wt) or with pUC19 (mock) were subjected to a 2-step purification. In the first step 5 ml sucrose cushions (20% in PBS) were overlaid with 35 ml of cell-free cell culture supernatant (0.45 μm sterile filtrate) and centrifuged for 90 min in SW-32Ti rotors at 25,500 rpm (~82,600 g_ave_) and 4°C. The pellets were then gently resuspended in 150 μl of PBS buffer per bucket (~200 fold volume concentration) and the content of 6 buckets of each supernatant type were pooled together. In the next step, 900 μl of pooled concentrated supernatants were successively loaded in 450 μl aliquots on Amicon Ultra 0.5 ml 100 K concentrators and centrifuged at 14,000 g (~5 min) until a final volume of ~90 μl (~2000 fold concentration). Two 20 μl aliquots of the concentrated supernatant from virus producing (wt) and control cells (mock) were then separately loaded on NuPAGE Novex 4–12% Bis-Tris Midi Protein Gels (Thermo Fisher Scientific), separated by gel electrophoresis and visualized with Coomassie staining. The gel regions at around 70 kDa corresponding to PFV Gag were then excised as indicated in S4A Fig and *in-gel* digested with trypsin [81]. The resulting peptide mixtures were analysed by LC MS/MS on an Ultimate3000 nanoLC system interfaced on-line to a Q Exactive HF hybrid Quadrupole-Orbitrap mass spectrometer (both Thermo Fisher Scientific, Bremen, Germany). MS/MS spectra were matched using MASCOT v.2.2.04 (Matrix Sciences Ltd, London, UK) and MS Amanda v. 1.0.0.7503 [82] programs with 5ppm mass accuracy for precursor and fragments, and finally manually evaluated.

### Confocal microscopy

The analysis of the intracellular distribution of C-terminal mCherry-tagged Gag constructs and N-terminal eGFP-tagged PLK constructs using confocal microscopy was done as described previously [37]. Briefly, 293T cells were co-transfected with 1.7 μg of Gag-mCherry-encoding and 2 μg of eGFP-PLK encoding construct, using PEI-transfection method in 100 mm dishes as described above. One day post-transfection, cells were replated at a concentration of 3 x 10^5^ cells per well on poly-L-lysine coated cover slips in 12-well plates. At 48 h post-transfection the cells were washed with cold PBS, fixed with 3% paraformaldehyde, and the cell nuclei were stained with DAPI for 5 min. Finally the cells were covered with Mowiol. Confocal laser scanning images were obtained on a Leica SP5, using a Leica HC PL APO 40x 1.25 oil immersion objective. Fluorescence images were evaluated using ImageJ software. For quantitative analysis of co-localization at least 100 cells showing co-expression of individual Gag-PLK protein combinations were evaluated.

### Quantitative PCR analysis

Preparation of particle and cellular samples for qPCR analysis was performed as previously described [37, 44]. Primers, Taqman probes and cycling conditions for specific quantification of PFV genome, eGFP, or human GAPDH are summarized in S1 Table. All sample values obtained using a StepOne Plus (Applied Biosystems) qPCR machine were referred to a standard curve consisting of 10-fold serial dilutions of respective reference plasmids containing the target sequences (puc2MD9 for eGFP sequences, pczHSRV2 for viral genomic sequences, pCR2.1-TOPO-GAPDH for GAPDH sequences). All sample values included were in the linear range of the standard curves with a span from 10 to 10^9^ copies. The values for the DNA or RNA content of viral particle samples obtained by the qPCR analysis are expressed as percentage of the wt (generated by transfection of cells with pcoPG4, pcoPP, pcoPE and puc2MD9 or alternatively pczHSRV2). Determined RNA values of cellular samples were calculated as copies / ng total RNA and expressed as percentage of the wt.

### Integration site profile analysis

After infection of HT1080 target cells with various viral supernatants generated by transient transfection of different combinations of plasmids of the four-component PFV vector system into 293T cells, cellular genomic DNA was extracted one week later and PFV integration sites were amplified by linker-mediated PCR, as described previously [48]. Bioinformatic and statistic analyses of RefSeq genes, LADs, and gene density were as described [48].

### Yeast two-hybrid analysis

The initial large scale Y2H screen was performed at the Preclinical Target Development, and Genomics and Proteomics Core Facilities of the German Cancer Research Center, Heidelberg, using PFV Gag Y2H bait constructs pCGB-PG and pNGB-PG and a commercially available HeLa cDNA library (Clontech). For all other yeast two-hybrid analyses, the Y2H Matchmaker System based on the AH109 S. cerevisiae strain (Clontech) was used. Double-dropout medium was used for selecting the yeast transfected with both the plasmid containing GAL4 activation domain and the plasmid bearing GAL4 DNA-binding domain. Quadruple-dropout medium was used for selecting yeast in which GAL4 activity was present due to interactions of transfected proteins/peptides.

For production of transformation-competent yeast cells, a standard protocol entailing lithium acetate was used [83, 84]. Briefly, yeast overnight culture was set up by resuspending a few colonies, grown on complete-YPD agar plates, in 100 ml of YPD media and overnight incubation at 30°C. The main yeast culture was setup the following day by adding 400 ml of YPD media to the overnight culture and incubating the culture at 30°C until its optical density measured at 600 nm (OD_600_) reached a value of 0.6. A culture of the appropriate OD_600_ was pelleted at 3000 rpm for 8 min and the yeast pellet was washed once with 200 ml of 1x LiAc solution (0.1 M lithium acetate, 10 mM Tris-HCl pH 8.0, 1 mM EDTA) and once with 30 ml of 1x LiAc solution, before being resuspended in 1.5 ml 1x LiAc solution.

After 2 h of incubation at room temperature, 0.375 ml of sheared salmon sperm DNA (10 mg/ml, Ambion) and 2.275 ml of PEG solution (40% (w/v) PEG 3350, 0.1 M lithium acetate, 10 mM Tris-HCl pH 8.0, 1 mM EDTA) were added. 67 μl of competent yeast cell solution and 1.5 μg DNA of each Y2H construct were mixed and incubated overnight at 30°C. On the following day, the yeast was subjected to a 15 minute heat shock at 42°C, after which 15–20 μl of transfected yeast cells were plated on double-dropout (-Leu/-Trp) agar plates. The plates were incubated for three to four days at 30°C. Colonies growing on double-dropout plates were resuspended in a mixture of 55% H_2_O + 25% glycerol + 20% double-dropout medium and plated on quadruple-dropout (-Leu/-Trp/-Ade/-His) agar plates. Yeast growth was analyzed after two to four days incubation at 30°C.

## Supporting Information

S1 Fig Yeast two-hybrid analysis of PFV Gag-PLK interactions. Different variants of the PFV Gag protein (full length [FL] and indicated truncations) were tested for interaction with human [hPLK], mouse [mPLK] and rat PLK proteins [rPLK] or, where indicated, respective PBDs. PFV Gag was provided fused to the N-terminus [Gag-DB] or C-terminus [DB-Gag] of the GAL4 DB in combination with Tsg101- or PLK proteins fused to the C-terminus [AD-Prey] of the GAL4 AD. Presence and absence of interaction between each two partners is marked by either “+” or “-“, respectively; nd: not determined. Data of n = 2–6 independent experiments are summarized. (A+B) Results of PFV Gag C-terminal truncation mutant interaction with human and mouse PLK proteins. (C) Minimal Gag interaction domains for binding to PLK2 protein variants. iKD: inactive kinase domain; caKD: constitutively active kinase domain; iPBD: inactive polo-box domain.
(TIFF)

S2 Fig Functional analysis of PFV Pol STP motifs. (A) Schematic representation of full-length PFV Pol with protease (PR), reverse transcriptase (RT), RNase H (RH), integrase (IN) enzymatic domains and C-terminal S_960_-T_961_-P_962_ and S_1057_-T_1058_-P_1059_ motifs highlighted. Solid vertical arrow: primary Pol processing site; dashed vertical lines: Pol subdomain boundaries. (B) Different variants of the PFV Pol protein (full length Pol with enzymatically inactive PR domain [Pol-iPR]; integrase domain [IN]) were tested for interaction with human [hPLK] or, where indicated, respective PBDs. PFV Pol-iPR or IN was provided fused to the N-terminus (Pol-iPR-DB) or C-terminus (DB-Pol-iPR) of the GAL4 DB in combination with PLK proteins, Pol-iPR or IN fused to the N-terminus (Prey-AD) or C-terminus (AD-Prey) of the GAL4 AD. Presence and absence of interaction between each two partners is marked by either “+” or “-“, respectively. Data of n = 2–5 independent experiments are summarized. (C) PFV virions were produced by transient transfection of 293T cells with the four-component PFV vector system containing combinations of Gag and Pol variants as indicated. Titers of harvested viruses were determined by flow cytometry analysis of infected HT1080 target cells three days post-infection. The mean values and standard deviation for each supernatant were calculated from samples of cells infected with serial virus dilutions as described in Material and Methods. The values obtained using wt PFV Gag and Pol expression plasmids were arbitrarily set to 100%. Relative means and standard deviations normalized for Gag content (except uninfected) from independent experiments (n = 3) are shown. Differences between means of wt Gag and wt Pol containing virus and the individual mutants were analyzed by Welch’s t test (*, p<0.05). Absolute titers of wt supernatants ranged between 1.2 x 10^6^ and 1.6 x 10^6^ eGFP ffu/ml.
(TIFF)

S3 Fig Localization studies of ectopically-expressed, fluorescently-tagged PFV Gag and PLK proteins in fixed mammalian cells. 293T cells were transfected with eGFP-PLK-expressing constructs alone (left panels) or a combination of eGFP or eGFP-PLK and Gag-mCherry encoding expression constructs, as indicated above each panel of images. Forty-eight hours post-transfection, protein localization patterns were examined in fixed cells by confocal laser scanning microscopy (CLSM). Channels of the individual fluorescence micrographs are indicated on top, and the PLK variant used is indicated on the left. Data are representative of n = 2–5 independent experiments. (A) Localization patterns of eGFP-tagged PLK proteins (detected in eGFP-PLK channel) in mitotic and interphase cells transfected with the corresponding constructs. (B) Localization patterns of eGFP-tagged PLK and wt mCherry-tagged Gag proteins detected in corresponding channels in mitotic and interphase cells. (C) Localization of eGFP-tagged PLK and T225A Gag-mCherry in mitotic and interphase cells. (D) Localization patterns of wt mCherry-tagged Gag and various eGFP-tagged rPLK proteins detected in corresponding channels in mitotic cells. Scale bar: 10 μm. iKD: inactive kinase domain; caKD: constitutively active kinase domain; iPBD: inactive polo-box domain.
(PDF)

S4 Fig Mass spectrometric analysis of PFV Gag phosphorylation. (A) Coomassie staining of concentrated and purified, cell-free cell culture supernatants harvested from transfected 293T cells and separated by SDS-PAGE. Boxes with white dashed lines indicate gel regions at around 65–75 kDa corresponding to PFV Gag in supernatant lysates of cells transfected with PFV 4-component vector (wt) or respective mock transfected (mock) cells that were excised for proteolytic digest and mass spectrometric analysis. No PFV Gag derived peptides were detectable in mock supernatant lysates. Ø: empty lane; mwm: molecular weight standard (unstained Precision Plus Protein Standard, Biorad). (B) Extracted ion chromatogram for precursor ions with m/z 989.469 and 1016.125 corresponding to triply charged un- and mono-phosphorylated tryptic peptide aa 222 to 250 ATSTPGNIPWSLGDDNPPSSSFPGPSQPR of particle-associated Gag protein. Arrows indicate peaks corresponding to non-phosphorylated peptide and phosphorylated peptide pool. (C) High resolution fragmentation spectrum of singly phosphorylated peptide aa 222–250. Unique peaks corresponding to phosphorylated N-terminal 8-mer fragment are marked with a red box. Signals assigned to b(0)3 and b(4)-98 ions—water loss of non-phosphorylated aa 3-mer fragment 222-ATS-224 and characteristic phosphogroup loss of mono-phosphorylated aa 4-mer fragment 222-ATSTp-225 –suggest that the P-group could be located at T225.
(TIF)

S5 Fig Analysis of PFV wt and iSTP virions in single-round- and multiple-round infection experiments. (A) Replication-deficient PFV virions were produced by transient transfection of 293T cells with the four-component PFV vector system, containing either the wt Gag or T225A iSTP Gag variant. Titers of harvested viruses were determined by flow cytometry analysis of infected HT1080, human MRC-5 fibroblasts or immortalized primary mouse C57BL/6 embryonic fibroblasts (MEF) three days post-infection. The mean values and standard deviation for each supernatant were calculated from samples of cells infected with serial virus dilutions as described in Material and Methods. The values obtained using wt PFV Gag expression plasmids were arbitrarily set to 100%. Relative means and standard deviations not normalized for Gag content from independent experiments (n = 4–9) are shown. Differences between means of wt virus and the individual mutants were analyzed by Welch’s t test (**, p<0.01). Absolute titers of wt supernatants ranged between 1.2 x 10^6^ and 1.2 x 10^7^ eGFP ffu/ml. (B) Replication-competent PFV virions were produced by transient transfection of proviral expression vectors, containing either the wt Gag or one of the denoted iSTP Gag variants into 293T cells. Viruses were harvested two days post-transfection and Gag content normalized amounts of viral supernatants were used to infect MRC5 fibroblasts using a 1:100 dilution. Cell-free supernatants of infected MRC5 cultures were harvested at the time points indicated. Virus titers were determined by titration on HT1080 PLNE target cells and flow cytometry analysis one day post-infection. Virus titers of one representative experiment out of two are shown.
(TIF)

S6 Fig Biochemical characterization of the PFV production levels, particle release and nucleic acid contents in producer cells and released virions. Replication-deficient PFV virions were produced by transient transfection of 293T cells with the four-component PFV vector system, containing either the wt Gag (wt) or one of the denoted iSTP- (T225A, P226A) and pmSTP (T225E) Gag variants. As controls, viruses containing wt Gag in combination with Pol with inactive reverse transcriptase (iRT) or inactive integrase (iIN) domain or particles produced in the absence of PFV Env expression construct (ΔEnv) were produced. The mock control (mock) included cells transfected with pUC19 alone. (A) Representative Western blot analysis of viral particles (virus) purified from 293T cell culture supernatant by ultracentrifugation through 20% sucrose and 293T cell lysates (cell). PFV proteins were detected using polyclonal antibodies specific for PFV Gag (α-Gag) or PFV Env LP (α-LP), a mixture of hybridoma supernatants specific for PFV Pol PR/RT and IN (α-PR/RT + α-IN), or a commercial monoclonal antibody specific for GAPDH (α-GAPDH). Serial dilutions of the wt samples (wt; lanes 1–4) were quantified to determine their relative protein contents compared to other samples. The identity of the individual proteins detected is indicated on the right. (B) Viral particle release was determined by quantitative Western blot analysis of viral particle lysates. Mean values and standard deviations (n = 2) are shown as relative values compared to the wild type control and normalized for cellular expression levels. (C) Quantification of PFV vgRNA in virus producing cells (vgRNA cell) and released particles (vgRNA virus) and particle-associated vgDNA (vgDNA virus). Mean values and standard deviation (n = 2) are shown as relative values compared to the wt control. Cellular values were normalized to GAPDH levels, viral particle values were normalized for Gag content.
(TIF)

S7 Fig Statistical analysis of integration site data. P values of resulting pairwise comparisons are shown. RefSeq gene and LAD P values were determined using Fisher’s Exact Test, whereas gene density P values were calculated by Wilcoxon Rank Sum Test. WT-1, S224A-1, and T225A-1 infections were performed on the same day, whereas S224A-2 and T225A-2 infections were performed side-by-side on a separate day. WT-2 and WT-3 infections were performed on separate independent days. P values > 0.05 are highlighted in bold, italic type.
(TIF)

S1 Table qPCR Primer/probe sets. PFV: prototype foamy virus; LTR: long terminal repeat; EGFP: enhanced green fluorescent protein; GAPDH: Glyceraldehyde-3-phosphate dehydrogenase; fwd: forward; rev: reverse. ^a^ FAM: 6-carboxyfluorescein; HEX: hexachloro-fluorescein; BHQ1: Black Hole Quencher 1; BHQ2: Black Hole Quencher 2.
(PDF)
