# Correction: Interactions of Prototype Foamy Virus Capsids with Host Cell Polo-Like Kinases Are Important for Efficient Viral DNA Integration

**DOI:** 10.1371/journal.ppat.1005956

**Published:** 2016-10-10

**Authors:** Irena Zurnic, Sylvia Hütter, Ute Rzeha, Roger Helbig, Nicole Stanke, Juliane Reh, Erik Müllers, Martin V. Hamann, Tobias Kern, Gesche K. Gerresheim, Fabian Lindel, Erik Serrao, Paul Lesbats, Alan N. Engelman, Peter Cherepanov, Dirk Lindemann

Dr. Roger Helbig should be included in the author byline. He should be listed as the fourth author. At the time his contributions were performed, Dr. Helbig’s affiliation was: Institute of Virology, Medical Faculty “Carl Gustav Carus”; CRTD/DFG-Center for Regenerative Therapies Dresden, Technische Universität Dresden, Dresden, Germany. His current affiliation is the Laboratory of Epigenetics and Chromosome Biology, Department of Biomedical Research, Institute for Molecular Biology and Biotechnology, Foundation of research and technology Hellas (IMBB-FORTH), Ionnina, Greece. The contributions of this author are as follows: Conceptualization and Investigation. The correct citation is: Zurnic I, Hütter S, Rzeha U, Helbig R, Stanke N, Reh J, et al. (2016) Interactions of Prototype Foamy Virus Capsids with Host Cell Polo-Like Kinases Are Important for Efficient Viral DNA Integration. PLoS Pathog 12(8): e1005860. doi:10.1371/journal.ppat.1005860
