# Supplementary figures and images for: Interactions of Prototype Foamy Virus Capsids with Host Cell Polo-Like Kinases Are Important for Efficient Viral DNA Integration
Source: PLoS Pathog. 2016 Aug 31;12(8):e1005860. doi: 10.1371/journal.ppat.1005860 (PMC5006980; doi:10.1371/journal.ppat.1005860)

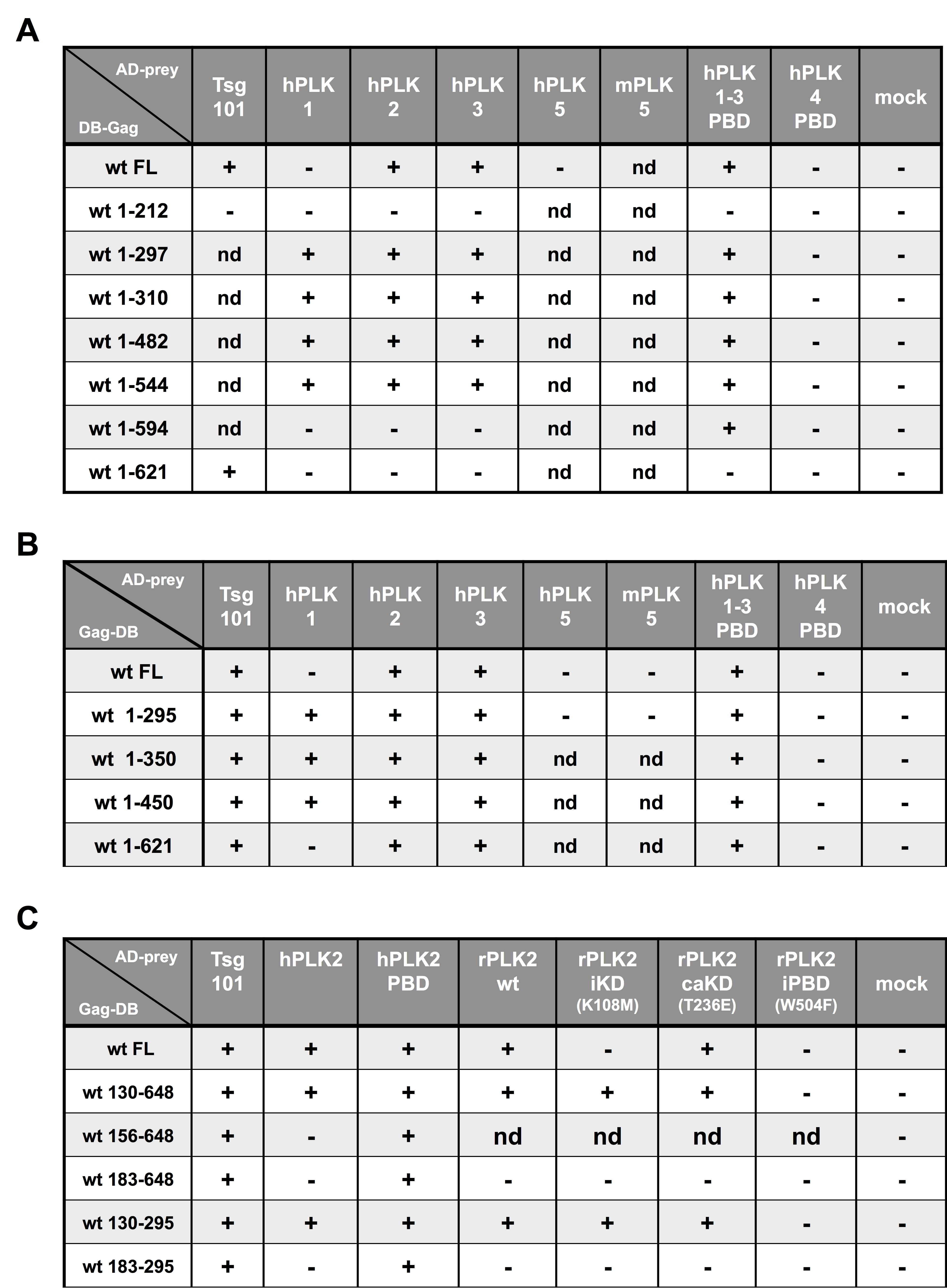

Supplement: S1 Fig — Different variants of the PFV Gag protein (full length [FL] and indicated truncations) were tested for interaction with human [hPLK], mouse [mPLK] and rat PLK proteins [rPLK] or, where indicated, respective PBDs. PFV Gag was provided fused to the N-terminus [Gag-DB] or C-terminus [DB-Gag] of the GAL4 DB in combination with Tsg101- or PLK proteins fused to the C-terminus [AD-Prey] of the GAL4 AD. Presence and absence of interaction between each two partners is marked by either “+” or “-“, respectively; nd: not determined. Data of n = 2–6 independent experiments are summarized. (A+B) Results of PFV Gag C-terminal truncation mutant interaction with human and mouse PLK proteins. (C) Minimal Gag interaction domains for binding to PLK2 protein variants. iKD: inactive kinase domain; caKD: constitutively active kinase domain; iPBD: inactive polo-box domain. (TIFF) [file ppat.1005860.s001.tiff]

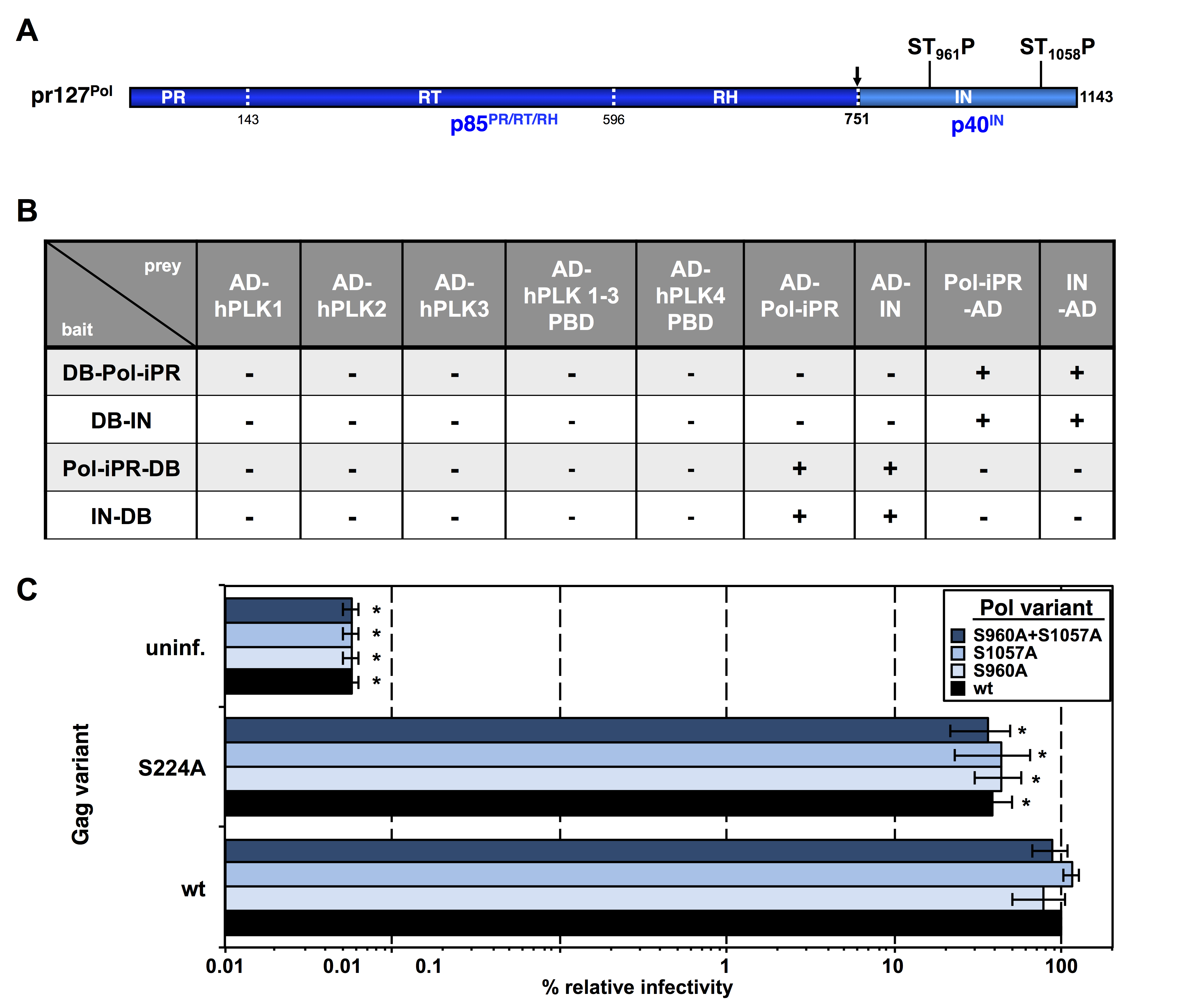

Supplement: S2 Fig — (A) Schematic representation of full-length PFV Pol with protease (PR), reverse transcriptase (RT), RNase H (RH), integrase (IN) enzymatic domains and C-terminal S960-T961-P962 and S1057-T1058-P1059 motifs highlighted. Solid vertical arrow: primary Pol processing site; dashed vertical lines: Pol subdomain boundaries. (B) Different variants of the PFV Pol protein (full length Pol with enzymatically inactive PR domain [Pol-iPR]; integrase domain [IN]) were tested for interaction with human [hPLK] or, where indicated, respective PBDs. PFV Pol-iPR or IN was provided fused to the N-terminus (Pol-iPR-DB) or C-terminus (DB-Pol-iPR) of the GAL4 DB in combination with PLK proteins, Pol-iPR or IN fused to the N-terminus (Prey-AD) or C-terminus (AD-Prey) of the GAL4 AD. Presence and absence of interaction between each two partners is marked by either “+” or “-“, respectively. Data of n = 2–5 independent experiments are summarized. (C) PFV virions were produced by transient transfection of 293T cells with the four-component PFV vector system containing combinations of Gag and Pol variants as indicated. Titers of harvested viruses were determined by flow cytometry analysis of infected HT1080 target cells three days post-infection. The mean values and standard deviation for each supernatant were calculated from samples of cells infected with serial virus dilutions as described in Material and Methods. The values obtained using wt PFV Gag and Pol expression plasmids were arbitrarily set to 100%. Relative means and standard deviations normalized for Gag content (except uninfected) from independent experiments (n = 3) are shown. Differences between means of wt Gag and wt Pol containing virus and the individual mutants were analyzed by Welch’s t test (*, p<0.05). Absolute titers of wt supernatants ranged between 1.2 x 10^6^ and 1.6 x 10^6^ eGFP ffu/ml. (TIFF) [file ppat.1005860.s002.tiff]

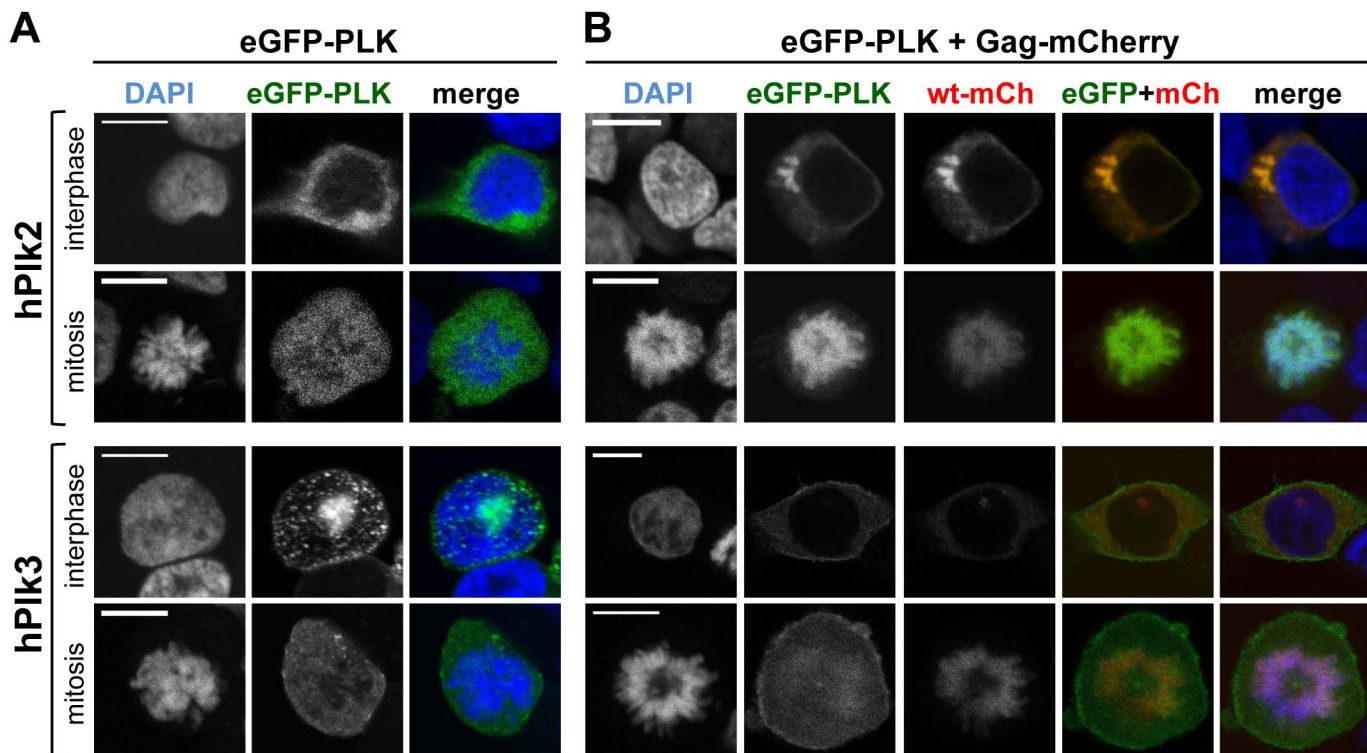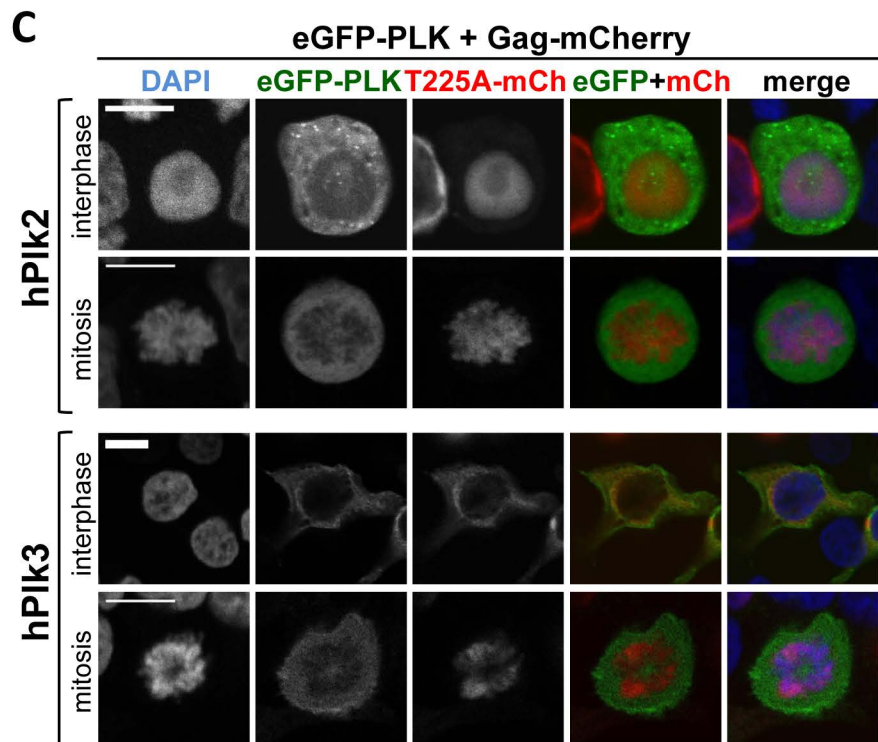

**D****eGFP-PLK + Gag-mCherry**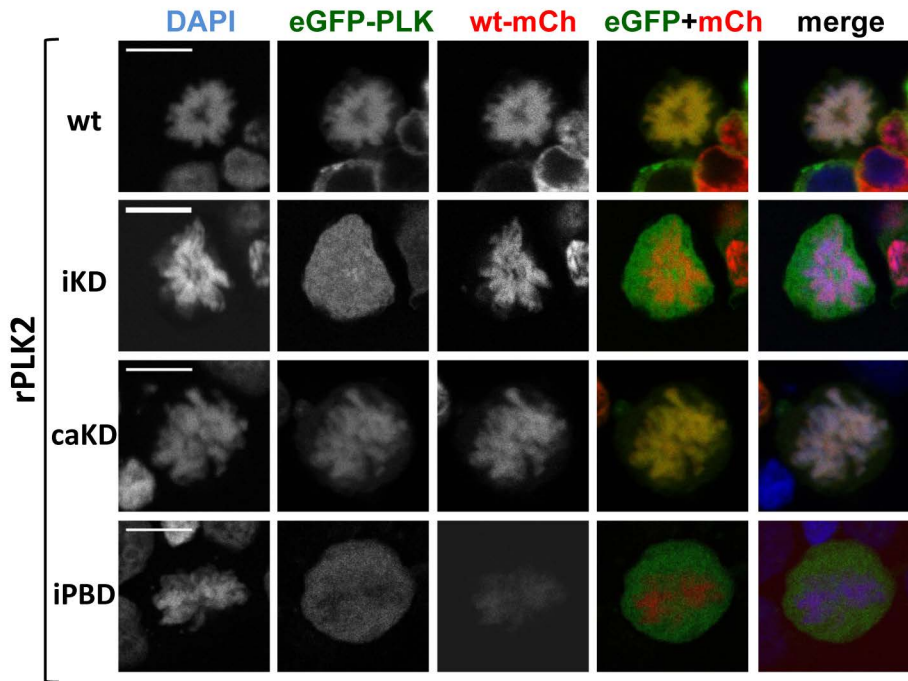

Supplement: S3 Fig — 293T cells were transfected with eGFP-PLK-expressing constructs alone (left panels) or a combination of eGFP or eGFP-PLK and Gag-mCherry encoding expression constructs, as indicated above each panel of images. Forty-eight hours post-transfection, protein localization patterns were examined in fixed cells by confocal laser scanning microscopy (CLSM). Channels of the individual fluorescence micrographs are indicated on top, and the PLK variant used is indicated on the left. Data are representative of n = 2–5 independent experiments. (A) Localization patterns of eGFP-tagged PLK proteins (detected in eGFP-PLK channel) in mitotic and interphase cells transfected with the corresponding constructs. (B) Localization patterns of eGFP-tagged PLK and wt mCherry-tagged Gag proteins detected in corresponding channels in mitotic and interphase cells. (C) Localization of eGFP-tagged PLK and T225A Gag-mCherry in mitotic and interphase cells. (D) Localization patterns of wt mCherry-tagged Gag and various eGFP-tagged rPLK proteins detected in corresponding channels in mitotic cells. Scale bar: 10 μm. iKD: inactive kinase domain; caKD: constitutively active kinase domain; iPBD: inactive polo-box domain. (PDF) [file ppat.1005860.s003.pdf]

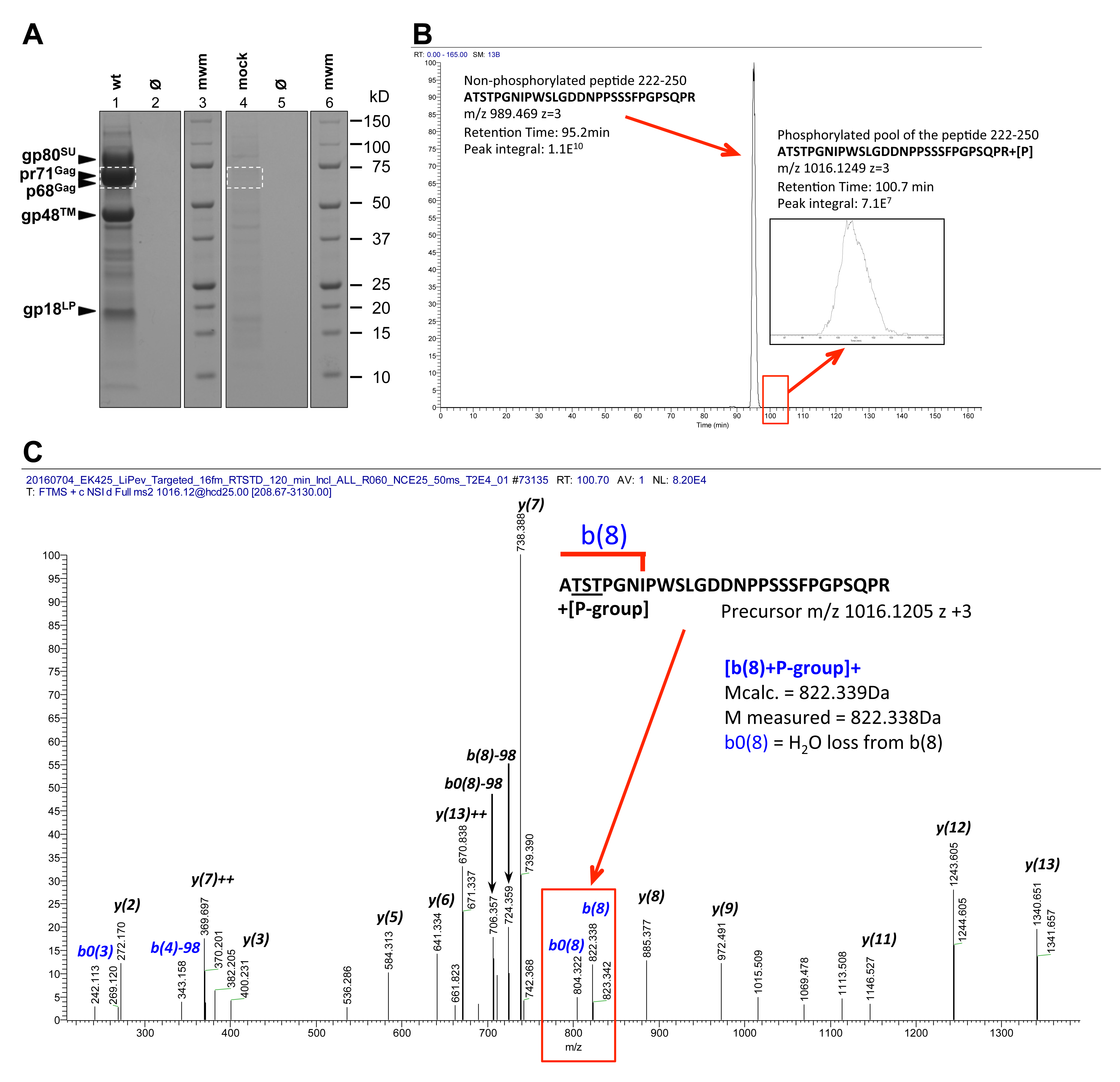

Supplement: S4 Fig — (A) Coomassie staining of concentrated and purified, cell-free cell culture supernatants harvested from transfected 293T cells and separated by SDS-PAGE. Boxes with white dashed lines indicate gel regions at around 65–75 kDa corresponding to PFV Gag in supernatant lysates of cells transfected with PFV 4-component vector (wt) or respective mock transfected (mock) cells that were excised for proteolytic digest and mass spectrometric analysis. No PFV Gag derived peptides were detectable in mock supernatant lysates. Ø: empty lane; mwm: molecular weight standard (unstained Precision Plus Protein Standard, Biorad). (B) Extracted ion chromatogram for precursor ions with m/z 989.469 and 1016.125 corresponding to triply charged un- and mono-phosphorylated tryptic peptide aa 222 to 250 ATSTPGNIPWSLGDDNPPSSSFPGPSQPR of particle-associated Gag protein. Arrows indicate peaks corresponding to non-phosphorylated peptide and phosphorylated peptide pool. (C) High resolution fragmentation spectrum of singly phosphorylated peptide aa 222–250. Unique peaks corresponding to phosphorylated N-terminal 8-mer fragment are marked with a red box. Signals assigned to b(0)3 and b(4)-98 ions—water loss of non-phosphorylated aa 3-mer fragment 222-ATS-224 and characteristic phosphogroup loss of mono-phosphorylated aa 4-mer fragment 222-ATSTp-225 –suggest that the P-group could be located at T225. (TIF) [file ppat.1005860.s004.tif]

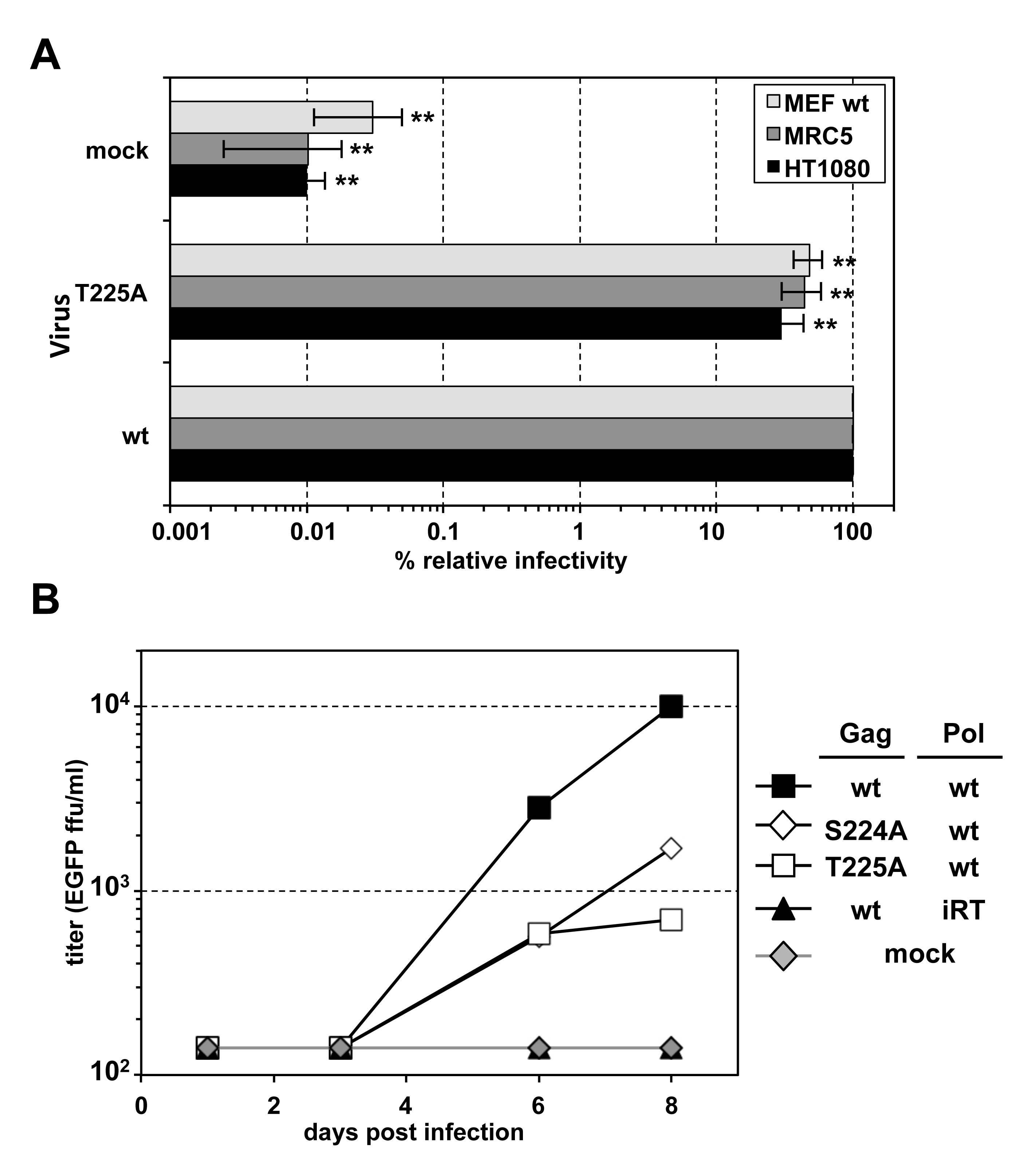

Supplement: S5 Fig — (A) Replication-deficient PFV virions were produced by transient transfection of 293T cells with the four-component PFV vector system, containing either the wt Gag or T225A iSTP Gag variant. Titers of harvested viruses were determined by flow cytometry analysis of infected HT1080, human MRC-5 fibroblasts or immortalized primary mouse C57BL/6 embryonic fibroblasts (MEF) three days post-infection. The mean values and standard deviation for each supernatant were calculated from samples of cells infected with serial virus dilutions as described in Material and Methods. The values obtained using wt PFV Gag expression plasmids were arbitrarily set to 100%. Relative means and standard deviations not normalized for Gag content from independent experiments (n = 4–9) are shown. Differences between means of wt virus and the individual mutants were analyzed by Welch’s t test (**, p<0.01). Absolute titers of wt supernatants ranged between 1.2 x 10^6^ and 1.2 x 10^7^ eGFP ffu/ml. (B) Replication-competent PFV virions were produced by transient transfection of proviral expression vectors, containing either the wt Gag or one of the denoted iSTP Gag variants into 293T cells. Viruses were harvested two days post-transfection and Gag content normalized amounts of viral supernatants were used to infect MRC5 fibroblasts using a 1:100 dilution. Cell-free supernatants of infected MRC5 cultures were harvested at the time points indicated. Virus titers were determined by titration on HT1080 PLNE target cells and flow cytometry analysis one day post-infection. Virus titers of one representative experiment out of two are shown. (TIF) [file ppat.1005860.s005.tif]

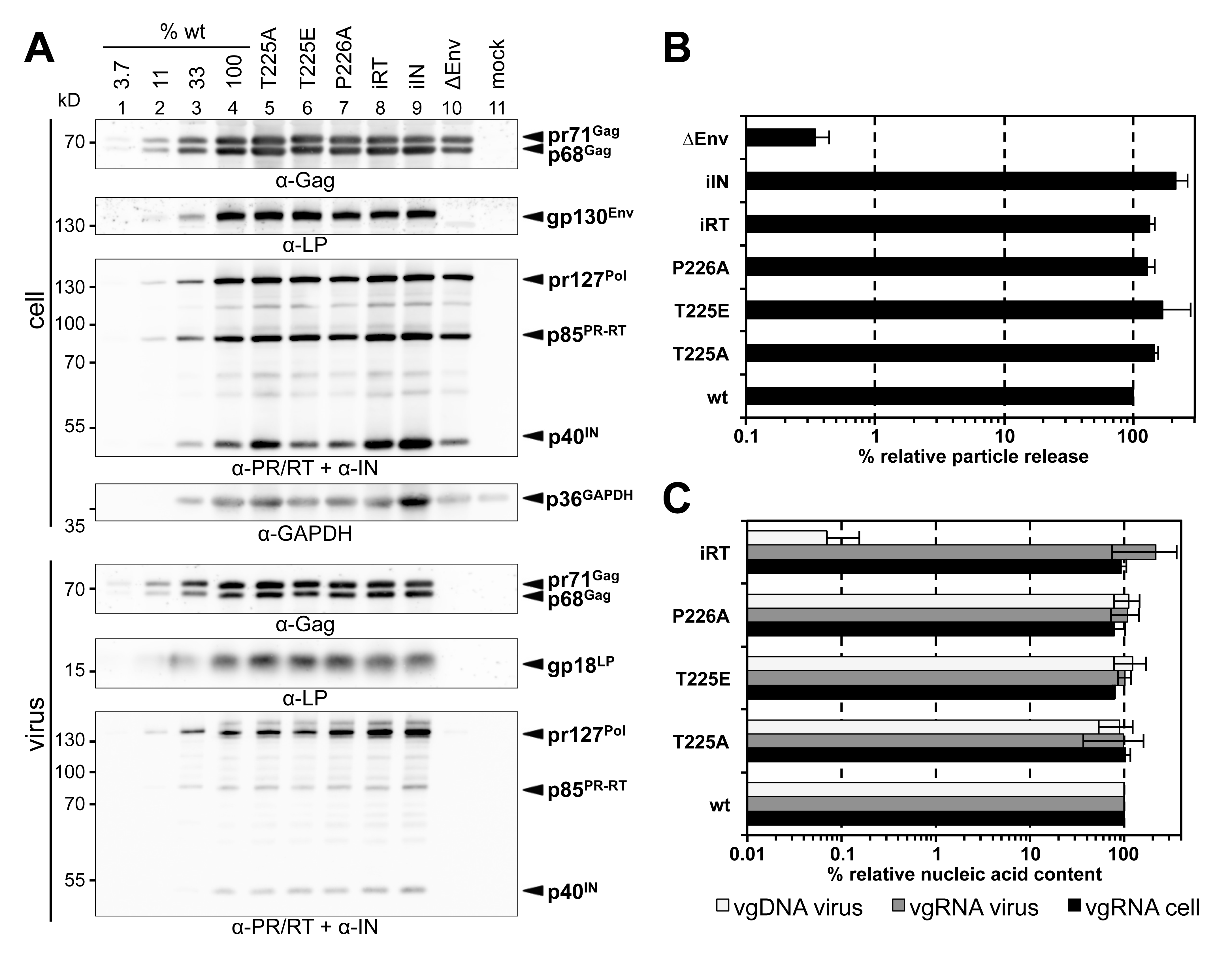

Supplement: S6 Fig — Replication-deficient PFV virions were produced by transient transfection of 293T cells with the four-component PFV vector system, containing either the wt Gag (wt) or one of the denoted iSTP- (T225A, P226A) and pmSTP (T225E) Gag variants. As controls, viruses containing wt Gag in combination with Pol with inactive reverse transcriptase (iRT) or inactive integrase (iIN) domain or particles produced in the absence of PFV Env expression construct (ΔEnv) were produced. The mock control (mock) included cells transfected with pUC19 alone. (A) Representative Western blot analysis of viral particles (virus) purified from 293T cell culture supernatant by ultracentrifugation through 20% sucrose and 293T cell lysates (cell). PFV proteins were detected using polyclonal antibodies specific for PFV Gag (α-Gag) or PFV Env LP (α-LP), a mixture of hybridoma supernatants specific for PFV Pol PR/RT and IN (α-PR/RT + α-IN), or a commercial monoclonal antibody specific for GAPDH (α-GAPDH). Serial dilutions of the wt samples (wt; lanes 1–4) were quantified to determine their relative protein contents compared to other samples. The identity of the individual proteins detected is indicated on the right. (B) Viral particle release was determined by quantitative Western blot analysis of viral particle lysates. Mean values and standard deviations (n = 2) are shown as relative values compared to the wild type control and normalized for cellular expression levels. (C) Quantification of PFV vgRNA in virus producing cells (vgRNA cell) and released particles (vgRNA virus) and particle-associated vgDNA (vgDNA virus). Mean values and standard deviation (n = 2) are shown as relative values compared to the wt control. Cellular values were normalized to GAPDH levels, viral particle values were normalized for Gag content. (TIF) [file ppat.1005860.s006.tif]

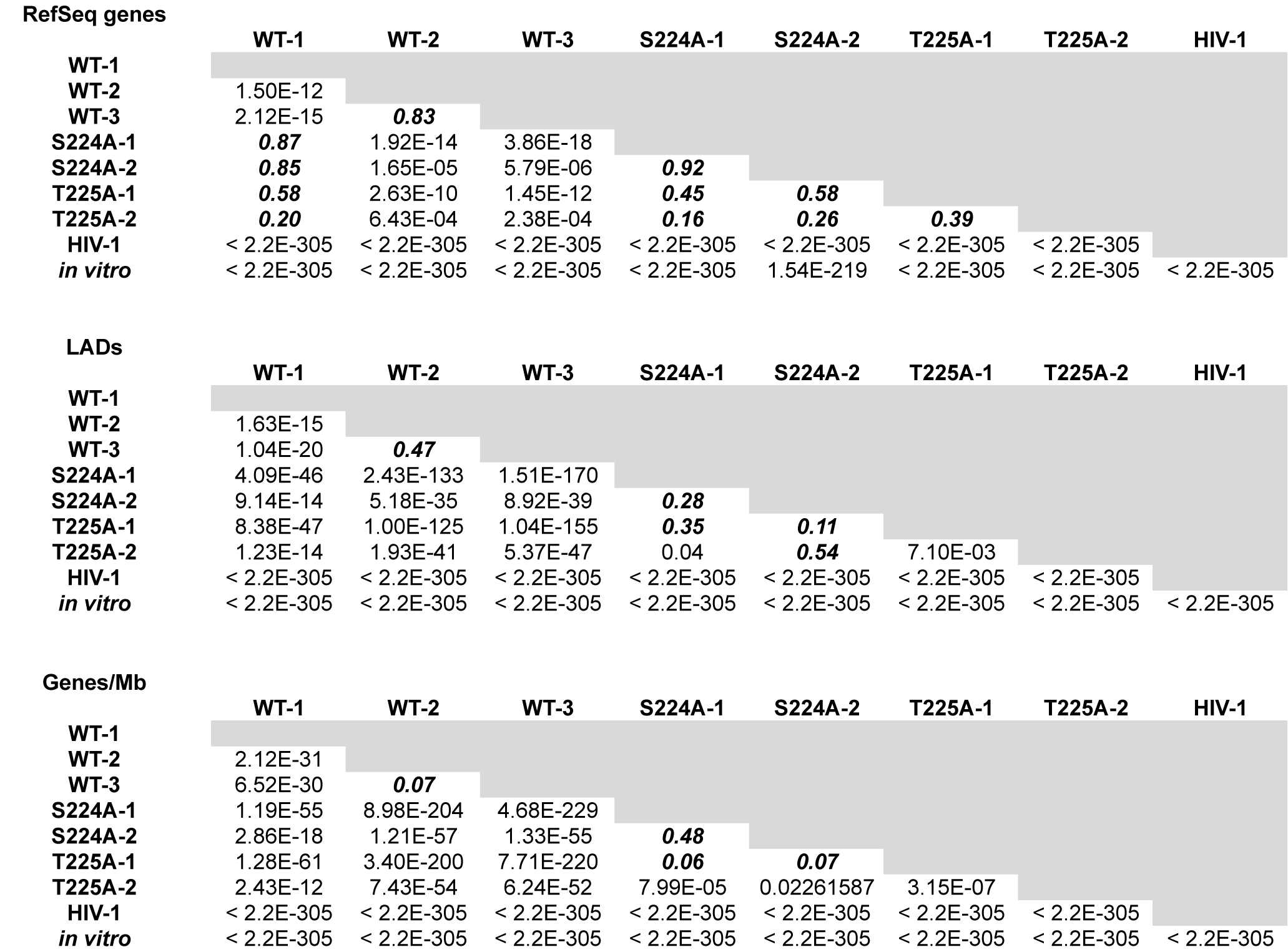

Supplement: S7 Fig — P values of resulting pairwise comparisons are shown. RefSeq gene and LAD P values were determined using Fisher’s Exact Test, whereas gene density P values were calculated by Wilcoxon Rank Sum Test. WT-1, S224A-1, and T225A-1 infections were performed on the same day, whereas S224A-2 and T225A-2 infections were performed side-by-side on a separate day. WT-2 and WT-3 infections were performed on separate independent days. P values > 0.05 are highlighted in bold, italic type. (TIF) [file ppat.1005860.s007.tif]
