# Supplementary material for: Interactions of Prototype Foamy Virus Capsids with Host Cell Polo-Like Kinases Are Important for Efficient Viral DNA Integration
Source: PLoS Pathog. 2016 Aug 31;12(8):e1005860. doi: 10.1371/journal.ppat.1005860 (PMC5006980; doi:10.1371/journal.ppat.1005860)
Supplement: S1 Table — PFV: prototype foamy virus; LTR: long terminal repeat; EGFP: enhanced green fluorescent protein; GAPDH: Glyceraldehyde-3-phosphate dehydrogenase; fwd: forward; rev: reverse. a FAM: 6-carboxyfluorescein; HEX: hexachloro-fluorescein; BHQ1: Black Hole Quencher 1; BHQ2: Black Hole Quencher 2. (PDF) [file ppat.1005860.s008.pdf]

Target	Primer/Probe	5'-3' Sequence[a]	Cycle Conditions
PFV genome (LTR R-U5)	fwd	TAAGGGTGATTGCAATGCTT	95 °C, 8 min, 1x
	rev	ATGTCTCCCTTAGCAAGGCT	95 °C, 30 s, 40x
	probe	FAM-TCAATAAACCGACTTGATTCGAGAACC-BHQ1	59 °C, 30 s, 40x
			72 °C, 30 s, 40x
EGFP	fwd	GCAGTGCTTCAGCCGCTAC	95 °C, 8 min, 1x
	rev	AAGAAGATGGTGCGCTCCTG	95 °C, 30 s, 40x
	probe	HEX-CCGACCACATGAAGCAGCACGACTT-BHQ2	59 °C, 30 s, 40x
			72 °C, 45 s, 40x
GAPDH	fwd	CATCAATGGAAATCCCATCA	95 °C, 8 min, 1x
	rev	GACTCCACGACGTACTCAGC	95 °C, 30 s, 40x
	probe	FAM-TCCAGGAGCGAGATCCCTCCA-BHQ1	59 °C, 30 s, 40x
			72 °C, 30 s, 40x
